# A sensory cell diversifies its output by varying Ca^2+^ influx‐release coupling among active zones

**DOI:** 10.15252/embj.2020106010

**Published:** 2020-12-21

**Authors:** Özge D Özçete, Tobias Moser

**Affiliations:** ^1^ Institute for Auditory Neuroscience and InnerEarLab University Medical Center Göttingen Göttingen Germany; ^2^ Collaborative Research Center 889 University of Göttingen Göttingen Germany; ^3^ Auditory Neuroscience Group Max Planck Institute of Experimental Medicine Göttingen Germany; ^4^ Göttingen Graduate Center for Neurosciences, Biophysics and Molecular Biosciences University of Göttingen Göttingen Germany; ^5^ Synaptic Nanophysiology Group Max Planck Institute of Biophysical Chemistry Göttingen Germany; ^6^ Multiscale Bioimaging Cluster of Excellence (MBExC) University of Göttingen Göttingen Germany

**Keywords:** calcium channel, exocytosis, nanodomain, synaptic heterogeneity, wide dynamic range coding, Neuroscience

## Abstract

The cochlea encodes sound pressures varying over six orders of magnitude by collective operation of functionally diverse spiral ganglion neurons (SGNs). The mechanisms enabling this functional diversity remain elusive. Here, we asked whether the sound intensity information, contained in the receptor potential of the presynaptic inner hair cell (IHC), is fractionated via heterogeneous synapses. We studied the transfer function of individual IHC synapses by combining patch‐clamp recordings with dual‐color Rhod‐FF and iGluSnFR imaging of presynaptic Ca^2+^ signals and glutamate release. Synapses differed in the voltage dependence of release: Those residing at the IHC' pillar side activated at more hyperpolarized potentials and typically showed tight control of release by few Ca^2+^ channels. We conclude that heterogeneity of voltage dependence and release site coupling of Ca^2+^ channels among the synapses varies synaptic transfer within individual IHCs and, thereby, likely contributes to the functional diversity of SGNs. The mechanism reported here might serve sensory cells and neurons more generally to diversify signaling even in close‐by synapses.

## Graphical abstract

Analysis of individual synapses reveal that the ability of auditory inner hair cells to translate a wide range of sound intensities is linked to presynaptic heterogeneity in Ca^2+^ channel‐mediated glutamate release.

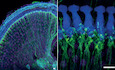

## Introduction

Neural systems employ functional diversity to achieve the complexity of behavior. Diversity is implemented at several levels, i.e., circuits, neurons, and subcellular functional units such as synapses. Sensory systems employ such multiscale diversity as well as adaptation to deal with the challenge of encoding a wide range of stimulus intensities (Kandel *et al*, 2012). The auditory system copes with processing a wide range of sound intensities by employing two types of sensory cells: outer hair cells to actively amplify and compress the range of mechanical inputs (Ashmore, 2008) and IHCs to adaptively encode at synapses with primary auditory neurons (type I spiral ganglion neurons [SGNs]) (Moser *et al*, 2019). Based on their physiology, these neurons can be classified into three functional subtypes, namely low, medium, and high spontaneous rate (SR) SGNs differing in the threshold and dynamic range of sound encoding (Kiang *et al*, 1965; Sachs & Abbas, 1974; Liberman, 1978; Winter *et al*, 1990; Taberner & Liberman, 2005). This functional SGN diversity appears at all tonotopic places of the cochlea, and the subtypes can even innervate the same IHC (Liberman, 1982).

Functional diversity of SGNs relates to the heterogeneity of their molecular profile, morphology, afferent, and efferent synaptic connectivity. In the cat (Liberman, 1982), back‐tracing experiments linked morphology to function and showed that low‐SR SGNs have thinner radial fibers (peripheral neurites) with fewer mitochondria than the high‐SR ones. Low‐SR SGNs preferentially innervate the modiolar (or neural) side of the IHC, where they face larger and more complex presynaptic active zones (AZs) (Liberman, 1980, 1982; Merchan‐Perez & Liberman, 1996; Kantardzhieva *et al*, 2013). Larger and more complex AZs at the modiolar side of IHCs were also found in mouse (Liberman *et al*, 2011; Ohn *et al*, 2016; Michanski *et al*, 2019), guinea pig (Furman *et al*, 2013; Song *et al*, 2016), and gerbil (Zhang *et al*, 2018). In the mouse, RNA sequencing of individual SGNs indicated three distinct molecular profiles (Sun *et al*, 2018; Shrestha *et al*, 2018; Petitpré *et al*, 2018) that were suggested to correspond to low, medium, and high‐SR SGNs based on the spatial segregation of their IHC innervation.

In mouse IHCs, AZ size correlates with the number of Ca^2+^ channels (approximately 30 to 300) (Neef *et al*, 2018) and consequently with the maximal Ca^2+^ influx at the AZ (Frank *et al*, 2009; Ohn *et al*, 2016; Neef *et al*, 2018). Should the analogy to the innervation pattern in the cat cochlea apply, it is odd that Ca^2+^‐triggered glutamate release from modiolar AZs, with large size and Ca^2+^ influx, was to drive low‐SR SGNs. A possible solution to this conundrum came from the finding that Ca^2+^ influx at the modiolar AZs requires stronger depolarization than at the pillar ones (Ohn *et al*, 2016). In other words, Ca^2+^ channels at modiolar AZs would be mostly closed at the IHC resting potential and require stronger receptor potentials to activate, which could explain the low SR and high thresholds of their postsynaptic SGNs.

Whether and how such heterogeneous properties of presynaptic Ca^2+^ signaling relate to glutamate release and to SGN firing remains to be elucidated. Exocytosis of readily releasable synaptic vesicles (SVs) in mature mouse IHCs relates near‐linearly to Ca^2+^ influx when varying the number of open Ca^2+^ channels (Brandt *et al*, 2005; Wong *et al*, 2014; Pangrsic *et al*, 2015). Similar findings were found in mouse vestibular hair cells (Dulon *et al*, 2009). Hence, one would assume that the heterogeneous Ca^2+^ signaling propagates into a concomitant diversity of transmitter release. However, this remains to be studied at the single‐synapse level ideally for several AZs of a given IHC. In fact, a recent study of cerebellar synapses highlighted how differences in Ca^2+^ channel–release coupling diversify synaptic transfer (Rebola *et al*, 2019).

Here, we studied the synaptic transfer function and underlying Ca^2+^ dependence of release at individual IHC‐SGN synapses by combining IHC patch‐clamp with imaging of synaptic Ca^2+^ influx and glutamate release. To detect glutamate release, we utilized the fluorescent glutamate reporter iGluSnFR (Marvin *et al*, 2013) that we targeted to the postsynaptic SGNs. Our results suggest that IHCs vary the voltage dependence of Ca^2+^ channels as well as their control of release sites among their AZs. This likely enables IHCs to signal the information contained in the receptor potential into complementary neural channels for encoding the entire audible range of sound intensities.

## Results

### Optical detection of glutamate release at individual inner hair cell synapses

**Figure 1:**
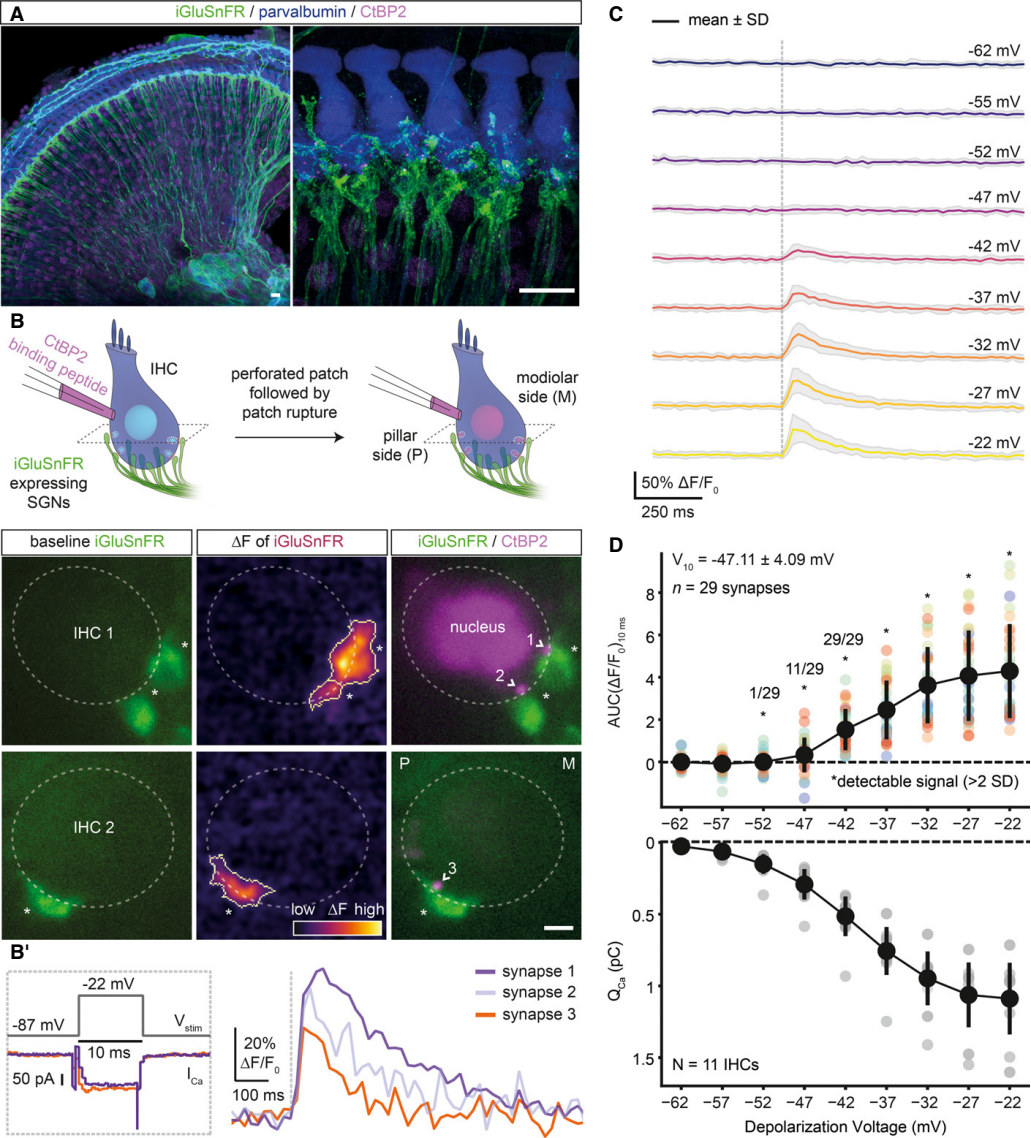
Optical detection of glutamate release at individual IHC synapses: low and variable voltage threshold AMaximum intensity projections of the organ of Corti from the right ear of a P17 mouse injected at P6 with 1–2 µl of AAV9.*hSyn*.iGluSnFR suspension, immunolabeled for iGluSnFR (GFP), IHCs, OHCs, and SGNs (parvalbumin) and synaptic ribbons and nucleus (CtBP2/RIBEYE). Close‐up (right panel) highlights IHCs innervated by several iGluSnFR‐expressing SGN boutons. Step sizes are 0.6 (left) and 0.5 µm (right), respectively. (Scale bars: 10 µm).BSimultaneous perforated patch‐clamp and imaging of IHCs (1.3 mM [Ca^2+^]_e_). IHCs were patch‐clamped from the pillar side, with a patch pipette containing TAMRA‐conjugated CtBP2‐binding peptide. Toward the end of the recording, the membrane patch was ruptured to fill the IHC with the peptide, which stains synaptic ribbons and nucleus. (Left) Two exemplary confocal sections of IHCs showing baseline fluorescence of iGluSnFR‐expressing afferent boutons from both modiolar (IHC1) and pillar (IHC2) side of the cell. Glutamate release from IHCs was evoked upon step depolarizations and detected as fluorescence change (ΔF) of iGluSnFR signal located on the SGN membrane (See (B′)). (Right) Overlaid images of the IHC1 and IHC2, displaying the boutons (iGluSnFR) and the synaptic ribbons (CtBP2), after the recording. (*: transduced afferent boutons, >: synaptic ribbons; Scale bar: 2 µm; see Appendix Fig S2 for the iGluSnFR‐ROI detection routine).B′The stimulus protocol of the example IHCs from (B), displaying the voltage stimulation (V_stim_), whole‐cell Ca^2+^ influx (I_Ca_) and single‐synapse iGluSnFR responses. IHCs were stimulated by 10‐ms‐long step depolarizations to −22 mV from the holding potential of −87 mV (1.3 mM [Ca^2+^]_e_), and iGluSnFR fluorescence was recorded at 50 Hz.CAverage ΔF/F_0_ iGluSnFR traces in response to 10‐ms‐long step depolarizations from the holding potential (−87 mV) to a voltage within the physiologically relevant range of receptor potentials: from −62 to −22 mV (applied in pseudo‐randomized order, step‐size 5 mV, perforated patch‐clamp, 1.3 mM [Ca^2+^]_e_, *n* = 29 boutons, *N* = 11 IHCs from nine mice). Shaded areas show ± SD.DThe voltage threshold of glutamate release was low and variable (−47.11 ± 4.09 mV, mean ± SD). The area under the curve of the iGluSnFR signal (top; AUC(ΔF/F_0_)_10ms_) from (C) and corresponding whole‐cell Q_Ca_ (bottom; mean ± SD). Detectable signals were defined here if the peak iGluSnFR signal was two times higher than baseline SD (depicted with *). All synapses had detectable signals in response to depolarizations ≥ −42 mV. (See also Figs EV1 and EV2).

Fluorescence imaging allows analysis of individual IHC AZs (Griesinger *et al*, 2005; Frank *et al*, 2009; Ohn *et al*, 2016) due to their large nearest neighbor distance (~ 2 µm) (Meyer *et al*, 2009). To image glutamate release, we targeted iGluSnFR (Marvin *et al*, 2013) to the postsynaptic SGN membrane. We injected the round window of WT mice at postnatal days (P)5–7 with adeno‐associated virus (AAV9, human synapsin promoter) to drive largely uniform SGN expression of iGluSnFR with several transduced afferent boutons per IHC (Fig 1A, Appendix Fig S1).

Using apicocochlear organs of Corti, acutely dissected after the onset of hearing (P15–19), we patch‐clamped IHCs and simultaneously imaged postsynaptic iGluSnFR fluorescence by spinning disk confocal microscopy (Ohn *et al*, 2016). Figure 1B shows two exemplary IHCs innervated by iGluSnFR‐expressing afferent boutons at their modiolar (IHC1) or pillar (IHC2) side in the given confocal sections. Sizable changes in iGluSnFR fluorescence (ΔF‐iGluSnFR) were evoked by brief (10‐ms‐long) step depolarizations to −22 mV (Fig 1B, see Appendix Fig S2 for the iGluSnFR‐region of interest [ROI] detection routine). Toward the end of the perforated patch recording, we ruptured the membrane patch and introduced a TAMRA‐conjugated dimeric ribbon‐binding peptide to identify individual AZs (Fig 1B). When imaging, we purposely avoided the basal cap of the IHCs in which separating individual postsynaptic boutons is more challenging given the high synapse density (Meyer *et al*, 2009; Liberman *et al*, 2011).

Next, we probed the effect of the imaging plane on the iGluSnFR signal. We applied 50‐ms‐long step depolarizations in seven different planes each separated from the next one by 0.5 µm (ruptured patch‐clamp, 10 mM intracellular EGTA, 5 mM [Ca^2+^]_e_, *n* = 10 boutons, *N* = 6 IHCs from four mice; Appendix Fig S3). We compared ΔF‐iGluSnFR in the optimal plane (the one with highest signal) to the ones in planes ± 0.5 µm and ± 1 µm from the optimal plane. We found that the ΔF‐iGluSnFR was rather robust toward missing the optimal plane: There was a 24.79 ± 4.33% reduction in the peak of the iGluSnFR signal for the “± 0.5 µm planes” and a 26.83 ± 4.05% reduction for the “± 1 µm planes”. Furthermore, we checked the variability of the ΔF‐iGluSnFR at a given synapse. We probed the peak of the iGluSnFR signal by repetitive 20‐ms‐long step depolarizations from the holding potential of −87 to −17 mV applied every 20 seconds over 5 min (Appendix Fig S4, ruptured patch‐clamp, 10 mM intracellular EGTA, 5 mM [Ca^2+^]_e_, *n* = 5 boutons, *N* = 2 IHCs from two mice). We observed a mild rundown of the ΔF‐iGluSnFR over repetitive stimulation: The mean peak ΔF amplitude of the last three points was 36.75 ± 7.03% smaller than the mean of the first three points (14 step depolarizations over 5 min).

**Figure EV1:**
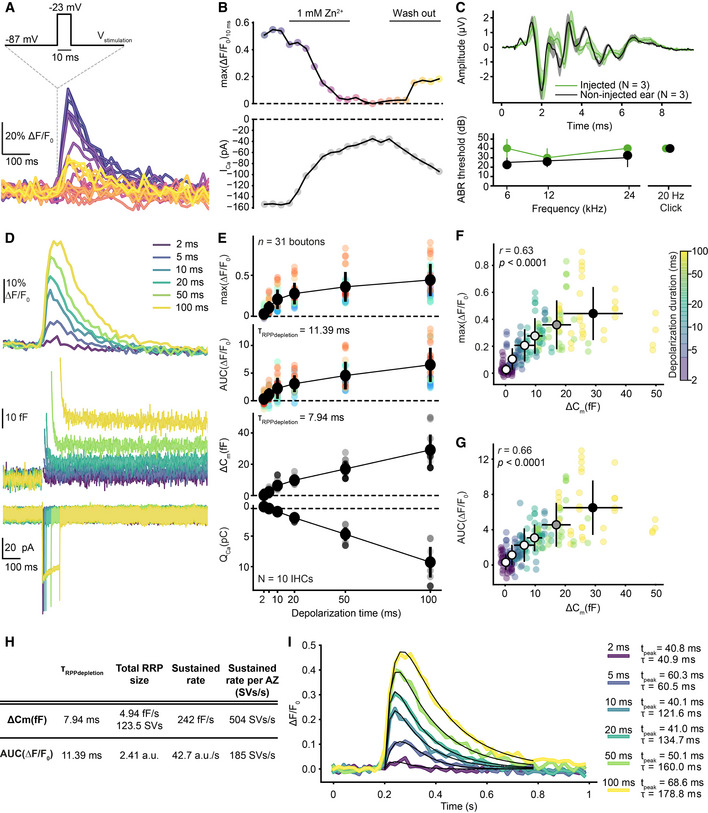
Characterization and validation of iGluSnFR for reporting IHC exocytosis. Related to Fig 1 AExemplary single‐synapse iGluSnFR signal in response to repetitive 10‐ms‐long step depolarizations to −23 mV from the holding potential (−87 mV) as Ca^2+^ channel blocker Zn^2+^ (1 mM) is perfused in and out of the recording chamber. The temporal sequence of the recordings is encoded by color; darker colors indicate earlier time points as in panel (B) (top).BThe time course of the peak iGluSnFR response (top; max(ΔF/F_0_)_10ms_) from (A) and corresponding whole‐cell peak Ca^2+^ current (bottom; perforated patch, 1.3 mM [Ca^2+^]_e_). The whole‐cell Ca^2+^ influx decreases with the perfusion of Zn^2+^.C(top) ABR waveforms of P29 WT mice, injected with AAV9.*hSyn*.iGluSnFR virus at P6, were recorded in response to 80 dB clicks (mean ± SEM, three animals). The non‐injected ear was used as a control. (bottom) ABR thresholds of the injected ear and the non‐injected control were comparable. A statistical test was not applied due to the small sample size. The presence of iGluSnFR expression was confirmed by immunostainings after ABR recordings.D–GiGluSnFR signal as a readout of glutamate release increases with stimulus duration along with the IHC's capacitance change (ΔC_m_). (D) Average responses of iGluSnFR (top), whole‐cell C_m_ (middle), and Ca^2+^ currents (bottom) upon step depolarizations to −23 mV from the holding potential of −87 mV for durations from 2 to 100 ms (color coded). Recordings were done in organs of Corti of P15–19 WT mice injected with AAV9.*hSyn*.iGluSnFR virus (perforated patch‐clamp, 1.3 mM [Ca^2+^]_e_, *n* = 31 boutons, *N* = 10 IHCs from eight mice). (E) The peak and the AUC of iGluSnFR signal, corresponding whole‐cell ΔC_m_, and Q_Ca_ plotted as a function of depolarization duration (mean ± SD). (F, G) The relation of whole‐cell ΔC_m_ and the peak (F) or the AUC (G) of the iGluSnFR signal (mean ± SD, *n* = 31 boutons, *N* = 10 IHCs from 8 mice). Both the peak and the AUC of iGluSnFR response correlate with the whole‐cell ΔC_m_ (Pearson's *r* = 0.63, *P* < 0.0001 and *r* = 0.66, *P* < 0.0001, Student's *t*‐test, respectively). Depolarization duration is color coded, and the black outlined circles, indicating the means, darken with increasing depolarization duration.HQuantification of exocytosis by whole‐cell ΔC_m_ and single‐synapse iGluSnFR‐AUC. (See Materials and Methods).IThe kinetics of iGluSnFR signal. Average iGluSnFR responses (as shown in panel (D) top). Black lines indicate the results of fitting to the average traces per depolarization duration (see Materials and Methods). Time to peak and the decay time constants are obtained from these fits and depicted with the color codes of the depolarization durations.

To probe the specificity of ΔF‐iGluSnFR for reporting Ca^2+^‐mediated glutamate release, we tested the effect of the Ca^2+^ channel blocker Zn^2+^. ΔF‐iGluSnFR triggered by step depolarizations gradually decreased and partially recovered upon Zn^2+^ application and wash‐out, respectively (Fig EV1A and B). To assess potential adverse effects of iGluSnFR expression on auditory signaling, we recorded auditory brainstem responses (ABR) at P29 (~ 23 days after the AAV injection). ABR waveforms and thresholds of the injected and non‐injected (control) ears were comparable (Fig EV1C) despite the efficient transduction and iGluSnFR expression of SGNs. In conclusion, AAV‐mediated expression of iGluSnFR in SGNs is suitable for studying IHC glutamate release with high specificity and does not obviously alter auditory physiology.

### Deciphering synaptic glutamate release from IHC active zones

To compare IHC exocytosis on a single‐synapse versus whole‐cell level, we measured synaptic ΔF‐iGluSnFR simultaneously with well‐established whole‐cell membrane capacitance changes (ΔC_m_) (Moser & Beutner, 2000). To assess the sensitivity and saturation of ΔF‐iGluSnFR, we applied stimuli of different durations (2–100 ms, in pseudo‐randomized order) in near‐physiological conditions (perforated patch configuration, 1.3 mM extracellular Ca^2+^ concentration ([Ca^2+^]_e_); Fig EV1D). ΔF‐iGluSnFR became significant at 2 ms (*P* < 0.0001), while ΔC_m_ were detectable only at 5 ms (*P* = 0.004; Appendix Fig S5). To evaluate a potential saturation of iGluSnFR by glutamate release, we related both the peak and the area under the curve of the iGluSnFR signal (hereafter referred to as iGluSnFR‐AUC) to the corresponding ΔC_m_. Both measures showed a positive correlation with ΔC_m_ (Pearson's *r* = 0.63, *P* < 0.0001 and *r* = 0.66, *P* < 0.0001; Fig EV1F and G), indicating their robust report of exocytosis for depolarizations up to at least 100 ms. Furthermore, the decay time constant of iGluSnFR signal increased with depolarization duration (Fig EV1I). Different from postsynaptic ΔF‐iGluSnFR, ΔC_m_ also reports extrasynaptic exocytosis (Pangrsic *et al*, 2015), likely contributing to the sublinear relationship of both measures for longer stimuli.

As a complementary approach to validate ΔF‐iGluSnFR as a readout of exocytosis, we probed it by applying brief (10‐ms) step depolarizations from the holding potential (−87 mV) to −57 mV in 10 mV increments up to 23 mV (applied in pseudo‐randomized order; Appendix Fig S6) and simultaneously recorded ΔC_m_. Both the peak and the AUC of iGluSnFR signal positively correlated with the ΔC_m_ in the negative voltage range (from −57 to −17 mV; Pearson's *r* = 0.57, *P* < 0.0001, Pearson's *r* = 0.59, *P* < 0.0001, respectively) and in the whole recorded voltage range (from −57 to 23 mV; Pearson's *r* = 0.58, *P* < 0.0001, Pearson's *r* = 0.61, *P* < 0.0001, respectively). These findings corroborated our observation in Fig EV1F and G that iGluSnFR saturation is not a major concern under these conditions.

Next, we compared SV pool dynamics by synaptic iGluSnFR‐AUC versus whole‐cell ΔC_m_. To estimate the dynamics of RRP and sustained exocytosis, we fitted the sum of an exponential and a linear function. The resulting time constants of RRP depletion were 11.39 ms for iGluSnFR‐AUC and 7.94 ms for ∆C_m_ (Fig EV1E and H, and see Materials and Methods). By estimating an average AZ RRP of ~ 10 SVs from ∆C_m_ measurements, we obtained an average change in iGluSnFR‐AUC (0.23 arbitrary units) per SV. The sustained component of exocytosis amounted to 42.7 a.u./s (~ 185 SV/s per AZ) for the recorded synapses compared to 242 fF/s (504 SV/s per AZ; Fig EV1H). The faster rate derived from whole‐cell ΔC_m_ measurements likely reflects the contribution of extrasynaptic exocytosis (Pangrsic *et al*, 2015). Hence, ΔF‐iGluSnFR can detect smaller amounts of exocytosis than whole‐cell ΔC_m_ and, on average, reports similar SV pool dynamics for single AZs.

**Figure EV2:**
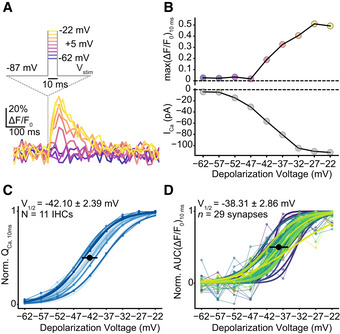
Low voltage threshold for IHC glutamate release. Related to Fig 1 Exemplary single‐synapse iGluSnFR signal in response to 10‐ms‐long step depolarizations from the holding potential of −87 mV to a voltage within the physiologically relevant range of receptor potentials: from −22 to −62 mV in 5 mV increments, same protocol as in Fig 1C and D.The peak iGluSnFR fluorescence (top; max(ΔF/F_0_)_10ms_) from (A) and corresponding whole‐cell Ca^2+^ current (bottom; perforated patch‐clamp, 1.3 mM [Ca^2+^]_e_). Glutamate release can be detected at −42 mV. The voltage range is color coded: Lighter points indicate more positive potentials.Normalized whole‐cell Q_Ca_, calculated in response to 10‐ms‐long step depolarizations, is plotted as a function of depolarization voltage (perforated patch‐clamp, 1.3 mM [Ca^2+^]_e_). Individual IHCs are color coded in shades of blue (mean ± SD, *N* = 11 IHCs from nine mice). A Boltzmann function was fitted to estimate the V_10_ and V_1/2_.Normalized iGluSnFR‐AUC, in response to 10‐ms‐long step depolarizations, same experiments as in (A) (mean ± SD, *n* = 29 synapses; individual synapses are color coded). A Boltzmann function was fitted to estimate the V_10_ and V_1/2_.

Finally, we studied how the RRP is recruited by brief graded depolarizations in the range of physiological receptor potentials (Russell & Sellick, 1983). Using near‐physiological conditions (perforated patch configuration, 1.3 mM [Ca^2+^]_e_), we applied 10‐ms‐long step depolarizations from the holding potential (−87 mV) to −62 mV in 5 mV increments up to −22 mV (applied in pseudo‐randomized order; Figs 1C and D, and EV2A and B). We analyzed the voltage dependence of glutamate release by fitting Boltzmann functions to iGluSnFR‐AUC (See Materials and Methods; Fig EV2C and D). The voltage threshold (V_10,_ defined as potential eliciting 10% of the maximum response) for glutamate release on average was −47.11 mV with considerable variance (SD: 4.09 mV; Fig EV2C and D). This is close to the threshold of activation of Ca_V_1.3 Ca^2+^ channels (~ −60 mV) (Zampini *et al*, 2010) and the *in vivo* resting membrane potential of IHCs (~ −55 mV) (Johnson *et al*, 2011). The variable voltage thresholds for glutamate release among the individual IHC synapses hint to differences in their transfer functions.

### Sequential dual‐color imaging of Ca^2+^ signal and glutamate release at single active zones

**Figure EV3:**
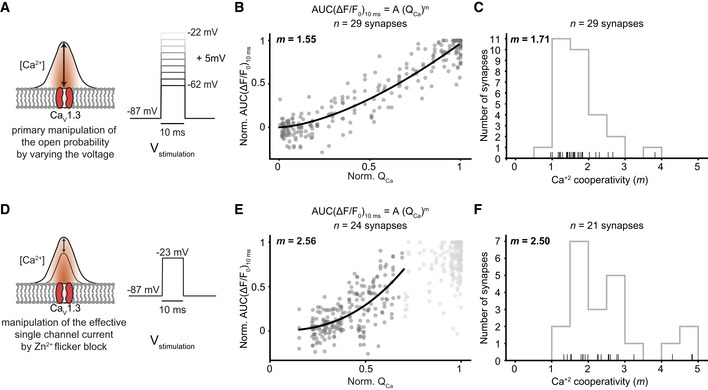
Relating release to whole‐cell Ca^2+^ influx during manipulation of open‐channel number or single‐channel current supports Ca^2+^ nanodomain‐like control of release. Related to Figs 1 and 3 Varying the voltage in the hyperpolarized range primarily varies the open‐channel number. RRP release was probed by 10‐ms‐long step depolarizations from the holding potential (−87 mV) to −62 to −22 mV in 5 mV steps applied in pseudo‐randomized order.The normalized AUC(ΔF/F_0_)_10ms_ is plotted versus Q_Ca_ (*n* = 29 boutons, *N* = 11 IHCs from nine mice): A power function was fitted before an obvious saturation of the RRP release, and a near‐linear relation was observed (*m* = 1.55).Histogram showing the distribution of *m* from individual fits per synapse before an obvious saturation of the RRP release (*m_avg_* = 1.71 ± 0.58). Only those fits with an *R* ^2^ value higher than 0.7 were used for further analysis (*n* = 29 boutons). The rug plot under the histogram displays individual data points. Every depolarization step was repeated at least two times per synapse, and the average was taken.Manipulation of the single Ca^2+^ channel current by Zn^2+^ perfusion. Note that Zn^2+^ acts as a rapid (microsecond) flicker blocker of Ca^2+^ channels (Winegar & Lansman, 1990), which, within the limits of IHC exocytosis kinetics (delay ~ 1 ms) (Beutner *et al*, 2001), is expected to reduce the fusogenic Ca^2+^ signal at the SV release site (Brandt *et al*, 2005). Therefore, this manipulation is expected to reveal the supralinear intrinsic Ca^2+^ dependence of release. We evoked RRP exocytosis by repetitive 10‐ms‐long step depolarizations to −22 mV, while slowly perfusing 1 mM Zn^2+^ into the recording chamber.Normalized AUC(ΔF/F_0_)_10 ms_ is plotted versus Q_Ca_ upon Zn^2+^ manipulation (*n* = 24 boutons, *N* = 10 IHCs from 10 mice). A power function was fitted before an obvious saturation of RRP release (normalized Q_Ca_ < 0.7), and a supralinear relation was observed (*m* = 2.56).Histogram showing the distribution of *m* from individual fits synapse before an obvious saturation was observed for a given synapse (*m*_average_ = 2.50 ± 1.03, *n* = 21 boutons with an *R* ^2^ of fit > 0.7). The rug plot under the histogram displays the individual data points.

How the opening of Ca_V_1.3 Ca^2+^ channels translates into glutamate release critically shapes synaptic transfer and is determined by the topography of Ca^2+^ channels and SV release sites. Previous studies evaluating the summed activity of several AZs indicate that a few channels in nanoscale proximity govern the [Ca^2+^] driving fusion of individual SVs at mature hair cell synapses (Ca^2+^ nanodomain‐like control of exocytosis) (Brandt *et al*, 2005; Graydon *et al*, 2011; Wong *et al*, 2014; Pangrsic *et al*, 2015). In the Ca^2+^ nanodomain‐like control of release, a near‐linear relation between release and Ca^2+^ influx is expected (apparent Ca^2+^ cooperativity of release (*m*) close to 1) when varying the number of open Ca^2+^ channels. Here, we found a near‐linear dependence (operationally defined as *m* < 2) of glutamate release at single AZs on the whole‐cell Ca^2+^ influx when depolarizing IHCs within the range of physiological receptor potentials (Fig EV3A–C, *m_QCa_* = 1.55, *n* = 29 synapses, *N* = 11 IHCs). Varying the voltage in this hyperpolarized range changes the open‐channel number, and for Ca^2+^ nanodomain control, this is more relevant than the change in single‐channel current as once the channel opens the ensuing Ca^2+^ signal tends to saturate the Ca^2+^ sensor of release (Pangrsic *et al*, 2015). In contrast, and consistent with the supralinear intrinsic Ca^2+^ dependence of exocytosis in IHCs (Beutner *et al*, 2001; Wong *et al*, 2014), reducing the effective single‐channel current by the rapid flicker‐block of Ca^2+^ channels by Zn^2+^ showed *m *> 2 (Fig EV3D–F, *m_QCa_* = 2.56, *n* = 24 synapses, *N* = 10 IHCs). Taken together, imaging of glutamate release as a function of whole‐cell Ca^2+^ influx corroborates the notion of a Ca^2+^ nanodomain‐like control of release in mature IHCs (Brandt *et al*, 2005; Goutman & Glowatzki, 2007; Wong *et al*, 2014; Pangrsic *et al*, 2015). However, the presynaptic heterogeneity of Ca^2+^ signaling in IHCs (Frank *et al*, 2009; Ohn *et al*, 2016) underscores the need of studying Ca^2+^ influx–release coupling at individual AZs.

**Figure 2:**
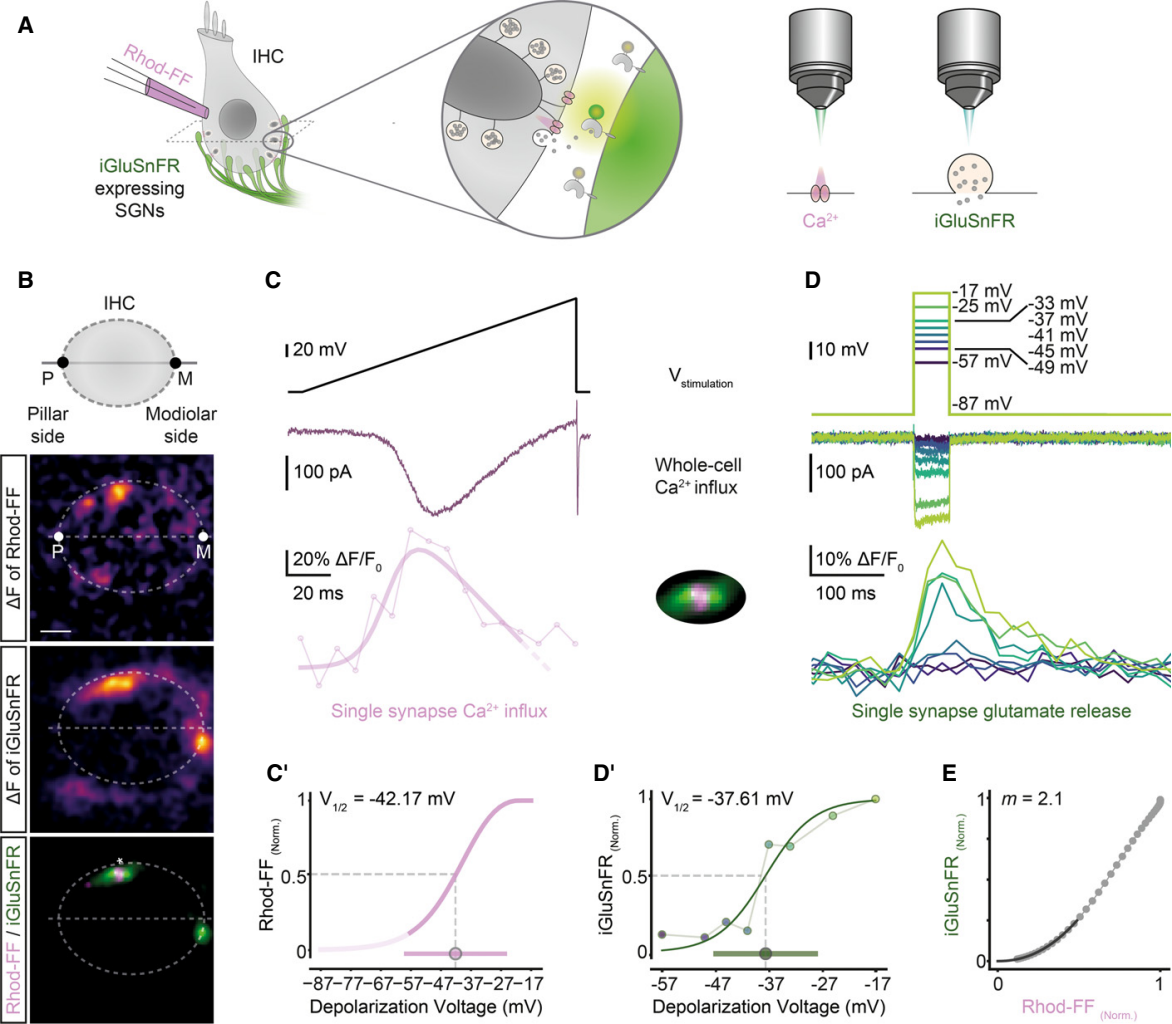
Sequential dual‐color imaging of synaptic Ca^2+^ influx and glutamate release AIHCs were patch‐clamped in ruptured‐patch configuration with 800 μM Rhod‐FF in the patch pipette, and simultaneously imaged for Ca^2+^ signals or glutamate release by spinning disk microscopy.BMean ΔF images of Rhod‐FF and iGluSnFR signals in response to a voltage ramp and a 50‐ms‐long step depolarization, respectively. The synapse marked with * on the overlaid image is further analyzed in the following panels. (P: pillar side, M: modiolar side; Scale bar: 2 μm)C, DVoltage command (top), corresponding whole‐cell Ca^2+^ influx (middle) and the functional fluorescence responses (bottom; band‐stop filtered at 33.3 Hz) from Rhod‐FF and iGluSnFR, respectively. A modified Boltzmann function (see Materials and Methods, *R* ^2^ = 0.81) was fitted to a Rhod‐FF fluorescence trace in response to a voltage ramp (C, C′). iGluSnFR‐AUC was calculated per depolarization voltage and used for a Boltzmann fit (D′, *R* ^2^ = 0.92). The voltage of half‐maximal activation (V_1/2_) and the dynamic range (10–90%) of synaptic Ca^2+^‐influx and glutamate release were calculated from the fits and depicted as circle and bar in (C′ and D′), respectively.EThe obtained fits from the Ca^2+^ “hot spot” (C′) and from glutamate release (D′) were plotted against each other in a voltage range from −57 to −17 mV in 1 mV increments. A power function was fitted up to the 25% of the maximum iGluSnFR‐AUC (*R* ^2^ = 0.99) to obtain the *m* estimate. (ruptured patch‐clamp, 10 mM intracellular EGTA, 5 mM [Ca^2+^]_e_; See also Fig EV4).

Studying the Ca^2+^ dependence of exocytosis at individual AZs has remained difficult. Previous studies related whole‐cell Ca^2+^ influx to either whole‐cell exocytosis (Brandt *et al*, 2005; Wong *et al*, 2014; Pangrsic *et al*, 2015) or to postsynaptic recordings of SV release (Goutman & Glowatzki, 2007). However, IHC synapses fundamentally vary in voltage dependence, number, and clustering of their Ca^2+^ channels (Frank *et al*, 2009; Meyer *et al*, 2009; Ohn *et al*, 2016; Neef *et al*, 2018). Here, we studied the operation of individual IHC synapses in the physiologically relevant voltage range during the 4^th^ postnatal week (P21–26). We combined ruptured patch‐clamp of IHCs with sequential dual‐color imaging of first synaptic Ca^2+^ signals and then glutamate release (Fig 2A). To image synaptic Ca^2+^ signals, we used the red‐shifted, low‐affinity (Kd:19 μM) chemical Ca^2+^‐indicator Rhod‐FF. We isolated Ca^2+^ signals at individual AZs using strong cytosolic buffering (10 mM EGTA in the patch pipette) and increased [Ca^2+^]_e_ (5 mM) (Frank *et al*, 2009; Ohn *et al*, 2016; Neef *et al*, 2018). We recorded glutamate release using ∆F‐iGluSnFR in SGN boutons contacting the mid‐section of IHC.

**Figure EV4:**
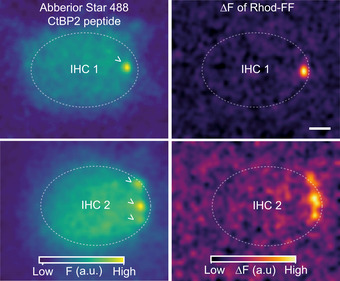
Depolarization‐evoked Ca^2+^ "hot spots" of Rhod‐FF fluorescence occur at the AZ. Related to Fig 2 (Left) Confocal sections of two example IHCs showing the fluorescence of the Abberior Star 488‐conjugated CtBP2‐binding peptide, which stains the synaptic ribbons. (Right) ∆F images of Rhod‐FF in response to a 100‐ms‐long step depolarization to −17 mV from the holding potential of −87 mV. The color maps of the F and ∆F images are in arbitrary units (a.u.) and displayed on the bottom. ( >: synaptic ribbons, scale bar: 2 µm).

Next, we studied the voltage dependence of synaptic Ca^2+^ influx and glutamate release. To probe the voltage dependence of synaptic Ca^2+^ influx, we imaged Rhod‐FF fluorescence (Fig 2B and C) while applying a voltage ramp (−87 to +63 mV), a fast protocol inducing a minimum cellular Ca^2+^ load (Ohn *et al*, 2016). This allowed us to analyze the Ca^2+^ influx of the AZ corresponding to a given SGN bouton, by repeating the voltage ramps in five different planes (separated by 0.5 μm). The hot spots of Rhod‐FF fluorescence elicited by depolarizations localized to the plasma membrane (Fig 2B) and to the synaptic ribbon (Fig EV4), indicating a cytosolic rise of [Ca^2+^] near the Ca^2+^ channel cluster of the AZ. Then, to probe the voltage dependence of glutamate release, we imaged iGluSnFR (in the central plane of Ca^2+^ imaging), while applying 50‐ms‐long step depolarizations (ranging from −57 to −17 mV) in a pseudo‐randomized fashion (Fig 2B and D). We used 50‐ms‐long depolarizations to elicit sufficient IHC release in the presence of 10 mM EGTA, which is expected to constrain the Ca^2+^ signal to the nanometer proximity of the Ca^2+^ channels and to slow Ca^2+^‐dependent SV replenishment (Moser & Beutner, 2000). Furthermore, the iGluSnFR‐AUC seemed robust toward saturation up to at least 100 ms of stimulation (see Fig EV1D–G and Appendix Fig S6). We analyzed the voltage dependences of synaptic Ca^2+^ influx and glutamate release by fitting Boltzmann functions to ∆F/F_0_ of Rhod‐FF (modified Boltzmann function, see Materials and Methods) and iGluSnFR‐AUC. In the example shown in Fig 2, the voltages of half‐maximal activation (V_1/2_) of Ca^2+^‐influx (Fig 2C′) and glutamate release (Fig 2D′) were −42.2 and −37.6 mV, respectively. The resulting fit functions were then used to relate the glutamate release and the synaptic Ca^2+^ influx over the physiologically relevant voltage range. To estimate the apparent Ca^2+^ dependence of release for individual synapses, we fitted a power function on this relation (Fig 2E). We restricted the fit until the 25% of maximum iGluSnFR‐AUC for all synapses, in order to avoid saturation, e.g., due to RRP depletion and obtained an *m* estimate (*m = *2.1 for the exemplary synapse). In conclusion, the sequential dual‐color imaging approach allowed us to study Ca^2+^ dependence of release at individual AZs.

### IHC synapses vary in voltage dependence and apparent Ca^2+^ dependence of release

**Figure 3:**
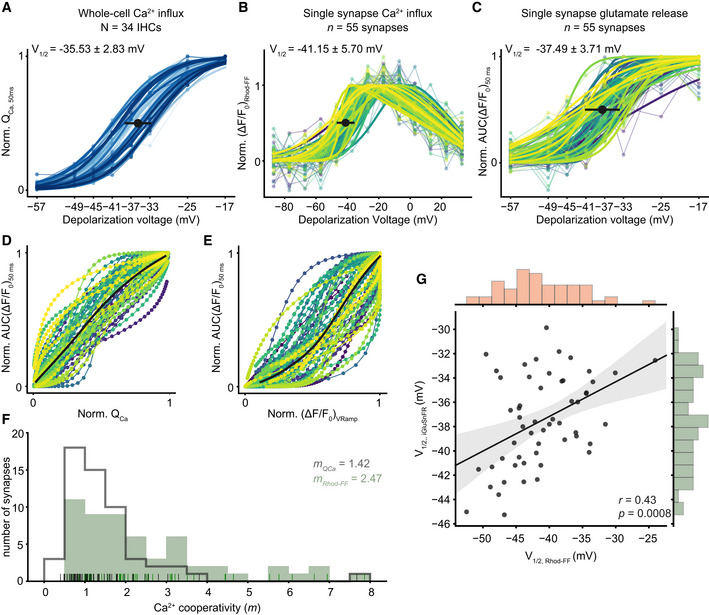
IHC synapses vary in voltage dependence and apparent Ca^2+^ dependence of release Normalized whole‐cell Q_Ca_, calculated in response to 50‐ms‐long step depolarizations, is plotted as a function of depolarization voltage. A Boltzmann function was fitted to estimate the V_1/2_. Individual IHCs are color coded in shades of blue (mean ± SD, *N* = 34 IHCs from 28 mice, ruptured patch‐clamp, 10 mM intracellular EGTA, 5 mM [Ca^2+^]_e_).A voltage ramp was applied to obtain ΔF of Rhod‐FF as a proxy of synaptic Ca^2+^ influx. The V_1/2_ of synaptic Ca^2+^ influx was calculated from a modified Boltzmann function (see Materials and Methods) fitted to ΔF/F_0_. (mean ± SD, *n* = 55 synapses; individual synapses are color coded; see Appendix Fig S7 for individual fits).Normalized iGluSnFR‐AUC, in response to 50‐ms‐long step depolarizations, same as (A) (see Appendix Fig S8 for individual fits).The relation of whole‐cell Ca^2+^‐influx (A) and the synaptic glutamate release (C).The relation of synaptic Ca^2+^ influx (B) and glutamate release (C). The bold lines show the means. (See also Appendix Fig S9 for individual plots)The histogram shows the Ca^2+^ cooperativities (*m*) obtained by individual power function fitting until 25% of normalized iGluSnFR response from ((D); gray) and from ((E); green). The mean *m* was found to be 1.42 and 2.47, respectively. The rug plot shows the individual data points.The V_1/2_ of synaptic Ca^2+^ influx and glutamate release is correlated (Pearson's *r* = 0.43, *P* = 0.0008, Student's *t*‐test). The marginal histograms show the distribution of each axis. Linear regression analysis (solid lines) and the associated 95% confidence intervals (shaded area). (See also Figs EV3, EV4, EV5).

**Figure EV5:**
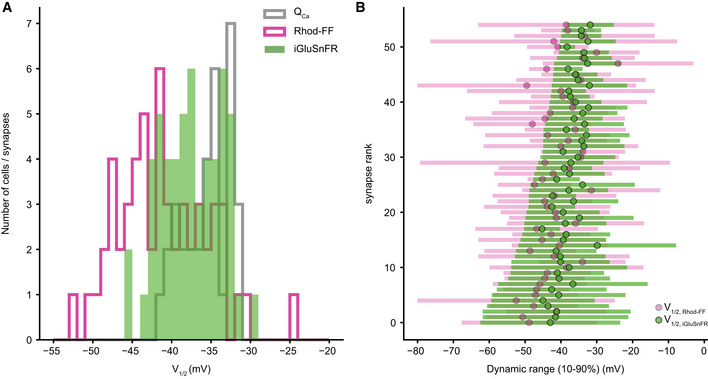
AZs vary in their voltage dependence. Related to Fig 3 V_1/2_ distribution of Q_Ca_ (gray, *N* = 34 IHCs), synaptic Ca^2+^ influx (magenta), and glutamate release (green, *n* = 55 synapses from 34 IHCs).Dynamic ranges (10–90%) of the synaptic Ca^2+^ influx (magenta) and glutamate release (green) with their V_1/2_ depicted. The synapses are ranked based on their glutamate release threshold (V_10_). Note how they gradually span the voltage range from −62.48 to −38.41 mV (mean ± SD = 48.27 ± 6.47 mV).

When systematically analyzing IHCs for the voltage dependence of whole‐cell Ca^2+^ influx (Q_Ca_), synaptic Ca^2+^ influx (∆F/F_0_ of Rhod‐FF), and glutamate release (iGluSnFR‐AUC), we observed fundamental heterogeneity of AZs within and across IHCs. The threshold for glutamate release was −48.27 ± 6.47 mV (SD, *n* = 55 synapses, *N* = 34 IHCs from 28 mice) and showed a broader distribution than that of the whole‐cell Ca^2+^ influx (V_10_ = −47.16 ± 3.19 mV, SD, *P* < 0.0001, Levene's test). As previously reported (Ohn *et al*, 2016), synaptic Ca^2+^ influx (V_1/2_ = −41.15 ± 5.70 mV, SD; *n* = 55 synapses, *N* = 34 IHCs from 28 mice, Fig 3B, see Appendix Fig S7 for individual fits) showed a broader and more negative V_1/2_ distribution than that of the whole‐cell Ca^2+^ influx (V_1/2_ = −35.44 ± 2.83 mV, SD, Fig 3A). The V_1/2_ distribution of synaptic glutamate release ranged from −45.25 to −29.86 mV and also showed a more negative average V_1/2_ (−37.49 ± 3.71 mV, SD, *n* = 55 synapses, *P = *0.008, Fig 3C, see Appendix Fig S8 for individual fits) than the whole‐cell Ca^2+^ influx. The V_1/2_ distributions differed significantly between glutamate release and synaptic Ca^2+^ influx (Fig EV5 and *P* = 0.009, Levene's test) as well as between synaptic and whole‐cell Ca^2+^ influx (*P* = 0.001, Levene's test). The V_1/2_ values of synaptic Ca^2+^ influx and glutamate release correlated (Pearson's *r* = 0.43, *P* = 0.0008, Fig 3G). This correlation indicates that the differences in the voltage dependence of Ca^2+^ influx are propagated to release, generating heterogeneous output among the IHC AZs at a given receptor potential.

Next, we estimated the apparent Ca^2+^ dependence of release by relating the iGluSnFR‐AUC to the whole‐cell Ca^2+^ influx (Fig 3D) and to the synaptic Ca^2+^ influx (Fig 3E) for the above voltage protocol that primarily varied the open‐channel number (see also Figs 1C and D, and EV3). The apparent Ca^2+^ dependence of glutamate release at the single‐synapse level (Fig 3F) was higher on average (*m_Rhod‐FF_* = 2.47, see Appendix Fig S9 for the data of individual IHCs) than when relating glutamate release to the whole‐cell Ca^2+^ influx (*m_QCa_* = 1.42). The observed discrepancy of the average *m* estimates from single‐synapse analysis and the *m* estimated based on the whole‐cell Q_Ca_ might at least partially be explained by the exclusion of synapses at the densely innervated basal IHC cap. We suspect that there are a fair number of synapses with *m < 2* in the basal cap. The *m* estimate based on the whole‐cell Q_Ca_ was lower on average than the *m* estimate obtained in the perforated‐patch configuration on P15–19 IHCs at 1.3 mM [Ca^2+^]_e_ using a similar stimulus protocol (see Figs 1C and D, and EV3A and C, *P = *0.001, Mann–Whitney *U*‐test). This difference is compatible with the hypothesis that strong Ca^2+^ buffering by 10 mM EGTA favors the operation of release sites under Ca^2+^ nanodomain control. Since the data of Fig EV3 were acquired at an earlier developmental stage (P15–19), the developmental tightening of the Ca^2+^ channel–exocytosis coupling (Wong *et al*, 2014) might also have contributed to the lower *m* in Fig 3D (P21–26). We did not find a significant difference between the thresholds of release with physiological buffering (perforated patch‐clamp, 1.3 mM [Ca^2+^]_e_) and with high EGTA buffering (ruptured patch‐clamp, 10 mM intracellular EGTA, 5 mM [Ca^2+^]_e,_ Mann–Whitney *U*‐test, *P* = 0.42), supporting the lack of obvious glutamate release quench in these high buffering conditions. Nevertheless, probing the synaptic Ca^2+^ dependence of release in more physiological buffering conditions remains important task for future studies. When operationally defining *m* < 2 (“near‐linear”) as indicative of Ca^2+^ nanodomain‐like control of release, we found approximately half of the synapses (29 out of 55 synapses) to operate in this scenario. The other half showed a broad spread of *m* values reaching up to 8, compatible with Ca^2+^ microdomain‐like control of release despite the presence of 10 mM EGTA. Taken together, single‐synapse imaging of Ca^2+^ influx and glutamate release revealed a heterogeneity of the apparent Ca^2+^ dependence of release that is likely due to differences in the coupling of Ca^2+^ channels to release sites among the IHC AZs. This heterogeneous coupling of Ca^2+^ channels to release sites likely contributes to the heterogeneous output of IHC AZs.

### Pillar synapses operate at more negative potentials than modiolar synapses

**Figure 4:**
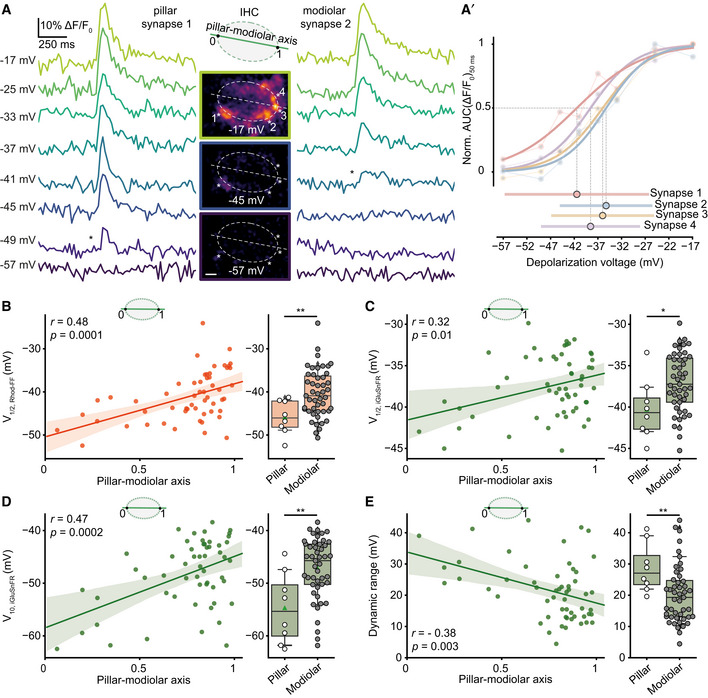
Pillar synapses are active at more negative potentials than modiolar synapses AThe single cell example shows release dynamics of two synapses innervating the same IHC from either pillar or modiolar side. iGluSnFR signals (band‐stop filtered at 33.3 Hz) in response to 50‐ms‐long step depolarizations to the given depolarization voltages are depicted (ruptured patch‐clamp, 10 mM intracellular EGTA, 5 mM [Ca^2+^]_e_). The middle panel shows ΔF images of iGluSnFR, recorded from the mid‐section of the IHC. * depict the first detected response in the given synapse. Note that pillar synapse 1 is already active at −49 mV, while modiolar synapse 2 starts responding only at −41 mV. (Scale bar: 2 μm).A′The normalized iGluSnFR‐AUC as a function of depolarization voltage. Dynamic range and V_1/2_ of the synapses are depicted in the lower panel.B–ELeft panel shows the linear regression analysis (solid lines) of V_1/2_ of synaptic Ca^2+^ influx (B), V^1/2^ (C), threshold (D), and dynamic range (E) of glutamate release as a function of position along the pillar–modiolar axis. Shaded areas depict the associated 95% confidence intervals. Significance of correlation coefficients is reported by a two‐tailed *P*‐value. Right panel shows box and whisker plots of these properties of synapses grouped into pillar and modiolar halves of the IHCs. Box plots indicate first quartile (25^th^ percentile), median and third quartile (75^th^ percentile) with whiskers reaching from 10 to 90%. For comparison of pillar and modiolar synapses, either Student's *t*‐test (for normally distributed data) or Mann–Whitney *U*‐test (for non‐normally distributed data) was applied. *P ≤ 0.05, **P ≤ 0.01.

SGNs exhibit a spatial preference in their IHC innervation pattern in the cat: High SR–low threshold fibers innervate the pillar side of the IHC, and low SR–high threshold fibers contact the modiolar side of the IHC (Liberman, 1982). Assuming analogy for mouse IHCs, we probed how presynaptic heterogeneity might contribute to the diversity of SGNs by analyzing the synaptic Ca^2+^ influx and glutamate release as a function of position along the pillar–modiolar axis. Figure 4A compares the simultaneous iGluSnFR responses of two exemplary synapses of one IHC: One positioned at the pillar and the other one at the modiolar side. In this example, the pillar synapse had a lower threshold; i.e., it already became active at −49 mV, while the modiolar synapse only started to respond at −41 mV. The pillar synapse also showed a more negative V_1/2_ and a wider dynamic range compared to the modiolar synapses in the given cell (Fig 4A′). As this observation was made in the same section of an IHC and was representative for the population (Fig 4B–E), we consider potential technical reasons unlikely. On average, V_1/2_ of both Ca^2+^ influx and glutamate release, and threshold of glutamate release (Fig 4B–D) were more negative at pillar synapses. Moreover, pillar synapses showed a wider dynamic range of release (Fig 4E). Nonetheless, there was substantial variability in particular among the modiolar synapses. This is obvious from the example shown in Fig 4A′ as well as at the population level (Fig 4B–E). We also performed linear regression analysis on the relation of pillar–modiolar position and V_1/2_ of synaptic Ca^2+^ influx as well as glutamate release (Fig 4B–E). This analysis confirmed a pillar–modiolar gradient of V_1/2_ of synaptic Ca^2+^ influx (*r* = 0.48; *P* = 0.0001) that we previously reported for an earlier postnatal stage (P14–20) (Ohn *et al*, 2016). Furthermore, it showed a pillar–modiolar gradient of the voltage threshold (*r* = 0.47; *p* = 0.0002), V_1/2_ (*r* = 0.32; *P* = 0.013), and dynamic range (*r* = −0.38; *P* = 0.003) of release. Ca^2+^ cooperativity did not show a significant correlation with the position along the pillar–modiolar axis (*r* = 0.17; *P* = 0.21; Appendix Fig S10). However, Ca^2+^ cooperativities ≥ 3 were mainly found for AZs on the modiolar side.

In conclusion, pillar and modiolar AZs differed in their voltage dependence and dynamic range of glutamate release. These differences between pillar and modiolar synapses support the hypothesis that diversity of the spontaneous and sound‐evoked SGN firing is, at least in part, rooted in the heterogeneous biophysical properties of presynaptic Ca^2+^ channels and transmitter release. Activation at more negative potentials of pillar synapses agrees with the high SR and low sound threshold of the corresponding SGNs. However, the wider dynamic range of glutamate release at pillar AZs is more difficult to reconcile with the narrower dynamic range of sound encoding of high SR–low threshold SGNs (Taberner & Liberman, 2005). While the exciting hypothesis of a modiolar–pillar segregation of synaptic properties has kept instructing important experiments, we should be aware of bias arising from this model. In fact, the present data seem to support a model that is between a strict ordering and a “salt‐and‐pepper” distribution of AZ properties in IHCs.

### Clustering of synaptic properties indicates three synapse subtypes likely distinguished by their implementation of Ca^2+^ influx–release coupling

**Figure EV6:**
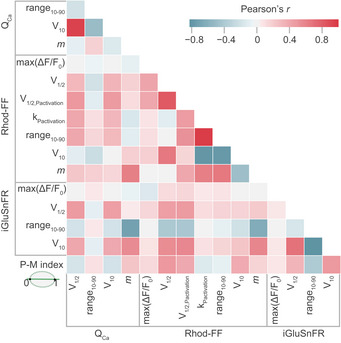
Correlation map of synaptic properties. Related to Fig 3 This correlation matrix shows the Pearson correlation coefficients (Pearson's *r*) between various properties assigned to individual synapses. The degree of correlation is color coded: light (weak) to dark (strong). Positive correlations are depicted in red and negative ones in blue.

**Figure 5:**
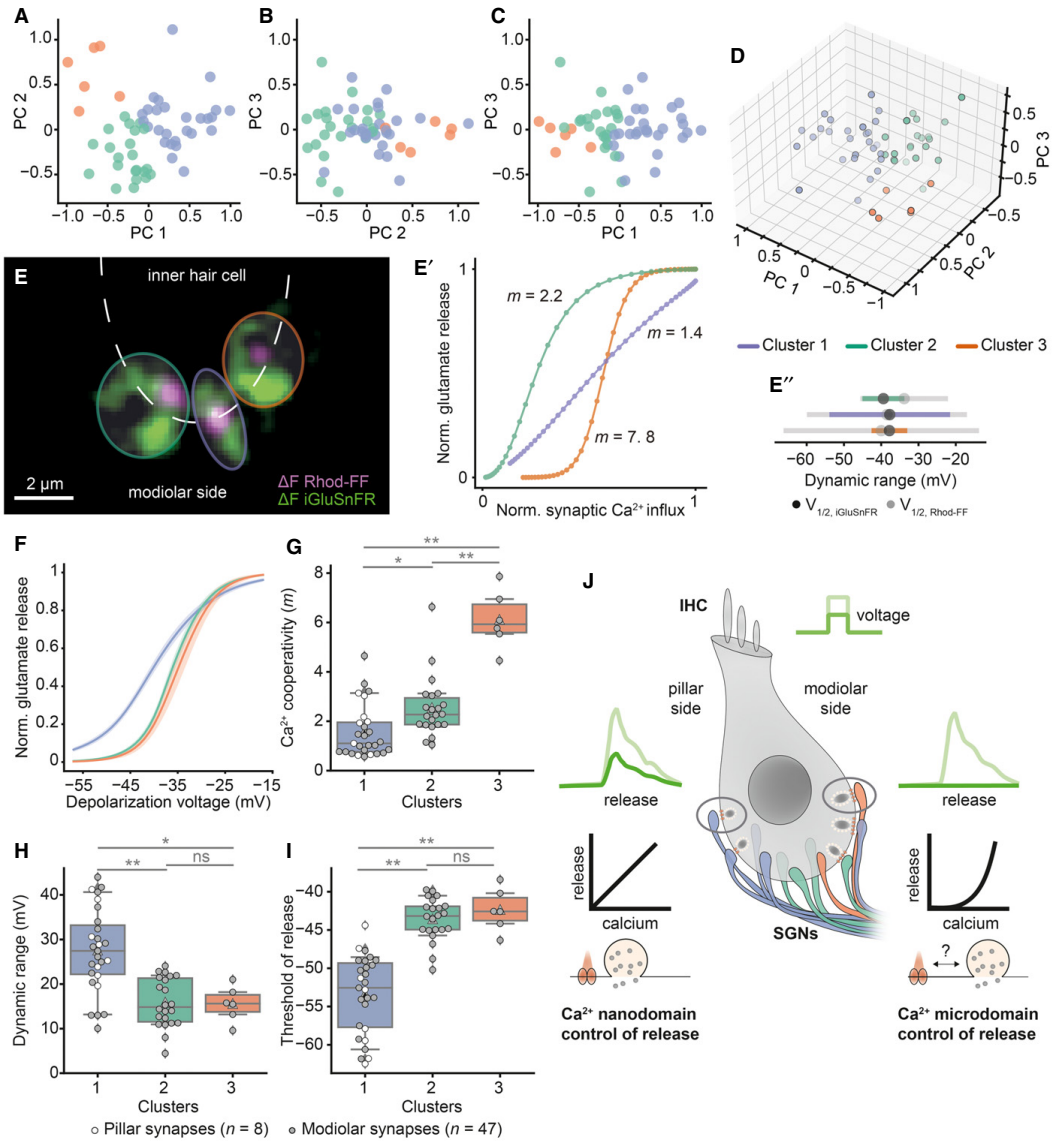
Three putative synapse subtypes, co‐existing in individual IHCs, differ in their synaptic transfer functions and Ca^2+^ dependencies 11 synaptic properties (except for the positional and whole‐cell information; see Fig EV6) of 55 synapses (*N* = 34 IHCs from 28 mice, ruptured patch‐clamp, 10 mM intracellular EGTA, 5 mM [Ca^2+^]_e_) were used for the PCA and *K*‐means clustering. A–CThe 2D plots of the first three principal components (PCs), labeled based on the clusters obtained by *K*‐means clustering.D3D plot showing the first three PCs.ESingle IHC exhibits different modes of Ca^2+^‐control of release. The overlaid mean ΔF image of Rhod‐FF (magenta) and iGluSnFR (green) shows synaptic Ca^2+^ influx and glutamate release at three neighboring modiolar synapses (individual synapses are color coded based on their clusters).E′The relation between Ca^2+^ influx and glutamate release of given synapses in the voltage range of −57 to −17 mV plotted with 1 mV increments. A power function was fitted until the 25% of normalized iGluSnFR‐AUC. Synapses showed different Ca^2+^ dependencies (*m* = 2.2, 1.4, 7.8; green, purple, orange).E″Dynamic ranges of corresponding Ca^2+^ influx (gray) and glutamate release (color coded) with V_1/2_ depicted. Note that the synapses from Cluster 1 (purple), employing Ca^2+^ nanodomain‐like control of release (*m* = 1.4), exhibit wider dynamic range than the other synapses.F–IMean glutamate release (iGluSnFR‐AUC) as a function of depolarization voltage in the three identified clusters (mean ± SEM, Cluster 1: *n* = 27 synapses, Cluster 2: *n* = 22 synapses, Cluster 3: *n* = 6 synapses). The Ca^2+^ cooperativity (*m*) (G), dynamic range (H), and glutamate release threshold (V_10_) (I) show differences for the three clusters. Cluster 1 is composed of linear synapses with wider dynamic range and lower threshold compared to the other clusters. Pillar synapses are depicted as white‐filled circles, and modiolar ones as gray‐filled circles. Box plots indicate first quartile (25^th^ percentile), median and third quartile (75^th^ percentile) with whiskers reaching from 10–90%. The clusters were compared by one‐way ANOVA test, followed by a *post hoc* Tukey's test. *P ≤ 0.05, **P ≤ 0.01.JThe proposed model for sound intensity encoding in an IHC. Differences in the presynaptic control of release, in terms of Ca^2+^ signaling and Ca^2+^ channel–exocytosis coupling, enable IHC to diversify the transfer functions of individual synapses for the same receptor potentials.

By the sequential dual‐color imaging of synaptic Ca^2+^ influx and release, we quantified each synapse with 15 parameters. Correlations among several of these parameters can be intuitively explained, such as the voltage dependence of a synapse, which is primarily rooted in that of Ca^2+^ channel activation (Fig EV6). Moreover, a supralinear coupling of release to Ca^2+^ influx is expected to compress the dynamic range of release. While the comparative analysis of pillar and modiolar synapses (see Fig 4) aims to elucidate synaptic correlates of the functional SGN properties, it has both value and limits. For an unbiased and in‐depth analysis, we applied *K*‐means clustering (*K* = 3) on the 11^th^‐dimensional space of single‐synapse properties (excluding positional and whole‐cell information) and obtained three synapse clusters (putative subtypes; see Appendix Fig S11 for clustering results with *K* = 2 and *K* = 4). To visualize the clusters in two or three dimensions, we performed principal component analysis (PCA) and used the first three principal components that explained 79% of the variance (Fig 5A–D). The main factors contributing to the first three PCs were the dynamic range of glutamate release, thresholds of glutamate release, and synaptic Ca^2+^ influx (Appendix Fig S12).

Next, we checked the response properties of these putative synapse subtypes. The first subtype of synapses showed a low *m* (1.56 ± 1.07, 27 synapses) indicating a Ca^2+^ nanodomain‐like control of release as well as a wide dynamic range (27.52 ± 9.37 mV) and a hyperpolarized activation threshold (−53 ± 5.07 mV) of release. In contrast, the third synapse subtype showed a high *m* (6.10 ± 1.18, six synapses) with smaller dynamic range (15.53 ± 3.95 mV) and a more depolarized threshold (−42 ± 2.8 mV; Fig 5F–I). Synapses from the first cluster are predicted to be active already around the IHC receptor potential (−55 mV, Fig 5I) (Johnson *et al*, 2011). The properties of the second synapse subtype fell in between those of subtypes 1 and 3. Finally, we evaluated positions of the three synapse subtypes in an effort to match them to the concept of a SGN‐subtype‐specific innervation pattern along the pillar–modiolar axis. Pillar synapses were exclusively of subtype 1, while modiolar synapses were distributed across all subtypes. This is exemplified by three neighboring modiolar synapses of an IHC (Fig 5E): Each synapse belongs to a separate cluster, employing different apparent Ca^2+^ dependencies (see Appendix Fig S9 for the data of all individual IHCs). Hence, different topographies of Ca^2+^ channels and SV release sites apparently co‐exist even in compact presynaptic cells. How the IHC manages to vary the AZ organization and, thereby, Ca^2+^ influx–exocytosis coupling remains to be elucidated. Our data indicate some extent of pillar–modiolar segregation of synaptic properties, while substantial heterogeneity is found among the AZ at all positions. Taken together, we propose a model in which IHCs fractionate the coding of sound intensity information, contained in the receptor potential, via heterogeneous synaptic input–output functions that are sourced by differences in voltage dependence and release site coupling to Ca^2+^ channels (Fig 5J).

## Discussion

The auditory system encodes sound pressures over a range of six orders of magnitude. The sensory mechanisms contributing to this fascinating behavior include active cochlear micromechanics, adaptation at various stages, as well as diversity of SGNs and their synapses with IHCs. At each tonotopic position in the cochlea, SGNs differ in their spontaneous and evoked firing, effectively tiling the range of audible sound pressures with their responses (Kiang *et al*, 1965; Sachs & Abbas, 1974; Liberman, 1978; Winter *et al*, 1990; Taberner & Liberman, 2005). Differences in the transcriptomic profiles of SGNs have led to their categorization into molecular subtypes (Sun *et al*, 2018; Shrestha *et al*, 2018; Petitpré *et al*, 2018). Moreover, heterogeneity in the pre‐ and postsynaptic properties of afferent IHC‐SGN synapses as well as of efferent SGN innervation have been proposed to explain the fractionation of the entire range of audible sound pressures, detected by the same IHC, into different neural representations (Ruel *et al*, 2001; Frank *et al*, 2009; Liberman *et al*, 2011; Ohn *et al*, 2016; Neef *et al*, 2018). Here, we studied the transfer at individual IHC‐SGN synapses, by imaging of synaptic Ca^2+^ signals and glutamate release during IHC patch‐clamp recordings. On average, glutamate release had a voltage threshold near the physiological resting potential (Figs 1 and 4) and showed a near‐linear dependence on the whole‐cell Ca^2+^ influx when primarily varying the open‐channel number (Fig EV3). We then employed sequential dual‐color imaging of Ca^2+^ signals and glutamate release of single AZs (Fig 2). This way we revealed the heterogeneity of synaptic transfer functions, rooted in differences in voltage dependence of the Ca^2+^ influx and in its coupling to exocytosis (Fig 3). We found a pillar–modiolar gradient of AZ transfer functions: consistent with the preferred innervation of high‐SR SGNs on the pillar side, pillar AZs operated at more negative potentials than the modiolar ones (Fig 4). By *K*‐means clustering of AZs according to the single‐synaptic parameters, we obtained three putative synapse subtypes, differing in the voltage dependence, apparent Ca^2+^ dependence, and dynamics of release (Fig 5).

### Heterogeneity of Ca^2+^ channel–release coupling

Studying Ca^2+^ signals and glutamate release at individual AZs revealed that the coupling of Ca^2+^ influx to exocytosis varies among the AZs, unlike previously assumed based on average AZ behavior (Brandt *et al*, 2005; Goutman & Glowatzki, 2007; Wong *et al*, 2014; Pangrsic *et al*, 2015). We show that it ranges from Ca^2+^ nanodomain to Ca^2+^ microdomain control of exocytosis and differs even among neighboring IHC AZs. Approximately one‐half of the AZs showed Ca^2+^ nanodomain‐like control of exocytosis (operationally defined as *m* < 2) during primary manipulation of the open‐channel number. Hence, different from what was proposed based on an AZ summation model (Heil & Neubauer, 2010), Ca^2+^ nanodomain‐like control of exocytosis occurs at individual IHC AZs. However, we found the other half of the AZs with *m> 2* suggesting Ca^2+^ microdomain control of exocytosis to co‐exist. We assume all AZs share the same intrinsic Ca^2+^ cooperativity of 4–5 that was established for IHC exocytosis by C_m_ recordings (Beutner *et al*, 2001; Brandt *et al*, 2005). This likely reflects the cooperative binding of 4–5 Ca^2+^ ions to the putative Ca^2+^ sensor of IHC release, otoferlin (Roux *et al*, 2006). In the 4^th^ postnatal week, we consider IHCs to have reached maturity but further changes might occur (Liberman & Liberman, 2016). However, the heterogeneity was preserved in all recorded ages (P21–26, Appendix Fig S13). Here, we studied synapses of apicocochlear IHCs and, given the previously described tonotopic differences in Ca^2+^ channel–release coupling on the whole‐cell level (Johnson *et al*, 2017), single‐synapse analysis in basocochlear IHCs remains an important task for the future. Taken together, we propose a model by which IHCs vary the topographies of Ca^2+^ channels and SV release sites at the AZs likely to diversify synaptic transmission beyond the heterogeneity of AZ size and Ca^2+^ signaling.

Non‐exclusive candidate mechanisms for position‐dependent AZ diversification in IHCs include (i) developmental competition of synapses, (ii) transsynaptic signaling from SGNs, and (iii) planar polarity signaling. One interesting idea is that, during development, pioneer SGN axons (Druckenbrod et al, 2020) making the first synaptic contact at the modiolar side of IHCs attract large amounts of presynaptic resources. In addition to such potential transsynaptic cueing of AZ properties, IHCs might employ their planar polarity signaling to instruct modiolar–pillar gradients of AZ size (Jean *et al*, 2019). How such signaling differentially shapes AZ properties remains an exciting topic for future studies. Clearly, polarized trafficking of components of the Ca^2+^ channel complex or other AZ proteins could contribute. This might, for instance, involve the adapter and PDZ‐domain protein Gipc3, defective in human deafness (Charizopoulou *et al*, 2011), required for the modiolar–pillar gradient of maximal synaptic Ca^2+^ influx (Ohn *et al*, 2016). Intriguingly, the spatial distribution of the number of Ca^2+^ channels and their voltage dependence might be regulated by different mechanisms (Jean *et al*, 2019). While the number of Ca^2+^ channels scales with AZ/ribbon size (Frank *et al*, 2009; Ohn *et al*, 2016; Neef *et al*, 2018) and shows a modiolar–pillar gradient, the topography relative to SV release sites and biophysical properties of the Ca^2+^ channels seem to follow an opposing gradient. For instance, the voltage dependence of synaptic Ca^2+^ influx (Ohn *et al*, 2016; Jean *et al*, 2019, and Fig 4) and, consecutively, glutamate release (Fig 4) shows a pillar–modiolar gradient, with activation at more hyperpolarized potential for the pillar AZs that show smaller Ca^2+^ channel clusters. Likewise, *m* of exocytosis was typically lower at the pillar side, indicating that Ca^2+^ nanodomain control of release prevails with the lower number of Ca^2+^ channels, while an increased channel number favors domain overlap as also predicted by modeling (Wong *et al*, 2014). Future studies will need to probe for differences in the abundance of potential molecular linkers (Han *et al*, 2011; Liu *et al*, 2011; Jung *et al*, 2015; Krinner *et al*, 2017) or spacers of Ca^2+^ channels and release sites such as RIM and RIM‐BP among the IHC AZs and to explore actual Ca^2+^ channels–release site topography of IHC AZs e.g. by electron microscopy (Nakamura *et al*, 2015).

As for size and Ca^2+^ channel number of the AZ, the positional dependence of biophysical properties and topography of Ca^2+^ channels might be shaped during postnatal development. For instance, AZs of immature IHCs, on average, employ a Ca^2+^ microdomain‐like control of exocytosis. Maturation tightens the coupling of Ca^2+^ influx and exocytosis at least when considering the collective behavior of all IHC AZs (Wong *et al*, 2014), coinciding with the appearance of high‐SR fibers (Wong *et al*, 2013). Our study indicates that a subset of mature IHC synapses employs Ca^2+^ microdomain control of release, representing an additional mechanism employed by the IHC to diversify synaptic transmission and endowing this subset with further potential of presynaptic plasticity (Vyleta & Jonas, 2014).

Potential pitfalls of our imaging approach to single IHC AZ function include the following: (i) glutamate spill‐over from neighboring synapses, (ii) saturation of iGluSnFR and other non‐linear effects resulting from the glutamate binding to endogenous receptors in the postsynapse, and (iii) contamination of synaptic Ca^2+^ signals by mitochondrial‐Ca^2+^ changes, as some rhodamine‐based dyes partition into mitochondria (Oheim *et al*, 2014). We aimed to minimize synaptic crosstalk by choosing well‐separated boutons. Moreover, simulations showed iGluSnFR provides a linear indication of glutamate release (Armbruster *et al*, 2020). While we cannot rule out a contribution of mitochondrial Rhod‐FF, the use of 10 mM EGTA would make mitochondrial‐Ca^2+^ uptake unlikely. This is supported with the strict co‐localization of the Rhod‐FF signals with the synaptic ribbon (Fig EV4).

### Relating presynaptic heterogeneity to functional SGN diversity

The functional subtypes of SGNs are said to spatially segregate their IHC innervation: High‐SR SGNs preferentially innervate the pillar side of the IHC, while low‐SR SGNs preferentially innervate the modiolar side of the IHC in the cat cochlea (Liberman, 1982). Here, we demonstrate by iGluSnFR imaging in the apical organ of Corti of mice that glutamate release from pillar synapses operates at more hyperpolarized potentials than the modiolar ones. This offers an exciting presynaptic hypothesis for the functional SGN diversity: Resting potential or weak receptor potentials will primarily recruit pillar synapses, which can readily explain the high SR and low sound threshold of SGNs innervating the pillar IHC side. While this hypothesis was previously phrased based on the heterogeneous voltage dependence of Ca^2+^ influx (Ohn *et al*, 2016), whether and how this translates into heterogeneity of glutamate release remained unclear. Indeed, unlike we had previously assumed (Ohn *et al*, 2016), the present study revealed differences in the Ca^2+^ channel–release coupling among the IHC AZs.

Heterogeneity of Ca^2+^ channel–release coupling, too, could contribute to diversifying SGN function. First, Ca^2+^ nanodomain control of exocytosis would increase the SR, as stochastic opening of Ca^2+^ channels could trigger release in such a tight coupling scenario (Moser *et al*, 2006; Eggermann *et al*, 2012). Interestingly, AZs of vestibular type I hair cells on average show very tight coupling of Ca^2+^ channels and exocytosis (Vincent *et al*, 2014) and are innervated by neurons with high spontaneous activity (~ 90 spikes/s) (Goldberg & Fernandez, 1971). Second, Ca^2+^ nanodomain control of exocytosis likely also promotes low voltage threshold for the same reason, and indeed, we found a correlation between the threshold of release and *m* (Fig EV6). Third, Ca^2+^ nanodomain control of exocytosis widens the dynamic range of release. Indeed, we found a negative correlation between dynamic range and *m* (Fig EV6). One of the most puzzling questions is how the pillar AZs with more hyperpolarized operation driving high‐SR SGNs that show a smaller dynamic range of sound encoding can be reconciled with the wider dynamic range of glutamate release found for pillar AZs. One possible explanation is high SR at the *in vivo* IHC resting potential lowers the standing RRP available for sound‐evoked release and hence causes a narrower dynamic range of release (reviewed in ref. (Moser *et al*, 2019)). Indeed, disruption of PDZ protein Gipc3 resulted in enhanced SR and narrower dynamic range (Ohn *et al*, 2016). Furthermore, as rate‐level functions are usually assessed in response to sound stimuli lasting more than 50 ms, there is likely a contribution of RRP replenishment (Pangrsic *et al*, 2015). We cannot exclude possible contribution of postsynaptic properties, efferent modulation, dynamic range adaptation (Wen *et al*, 2009), and non‐linearity imposed by the basilar membrane (Sachs & Abbas, 1974) on the dynamic range of SGN firing. Lastly, differences in Ca^2+^ channel–exocytosis coupling could affect the short‐term plasticity (reviewed in ref. (Böhme *et al*, 2018)), note the higher adaptation strength of high‐SR SGNs *in vivo* (Heil & Peterson, 2015). A similar example of linear and non‐linear synapses comes from the zebrafish bipolar cells (Odermatt *et al*, 2012) that encode light intensities over four orders of magnitude. In conclusion, we propose a model where differences among the IHC AZs in the presynaptic control of release, in terms of presynaptic Ca^2+^ signaling and Ca^2+^ channel–exocytosis coupling, enable a single IHC to diversify the synaptic signaling to SGNs for the same receptor potential. We suggest that non‐linear transformation of the sensory signal by heterogeneous synapses extends the dynamic range of intensity coding.

## Materials and Methods

### Reagents and Tools table

**Table:** 

Reagent/resource	Reference or source	Identifier or catalog number
**Experimental Models**		
C57BL/6J (*Mus musculus*)		
**Recombinant DNA**		
pAAV‐hSyn‐iGluSnFR	Marvin *et al* (2013)	RRID:Addgene_98929
**Antibodies**		
Chicken anti‐GFP	Abcam	Abcam Cat# ab13970, RRID:AB_300798
Guinea pig anti‐parvalbumin	Synaptic Systems	Cat# 195 004, RRID:AB_2156476
Mouse anti‐CtBP2	BD Biosciences	Cat# 612044, RRID:AB_399431
**Chemicals, enzymes and other reagents**		
Rhod‐FF, tripotassium salt	AAT Bioquest, Biomol	Cat #: 21075
TAMRA‐conjugated CtBP2 peptide	Zenisek *et al* (2004)	BioSynthan
Abberior Star 488‐conjugated CtBP2 peptide	Zenisek *et al* (2004)	BioSynthan, MPIbpc
Amphotericin B	Merck (Calbiochem)	Cat #: 171375
**Software**		
Python	Python Software Foundation	https://www.python.org/psf/
Igor	WaveMetrics	https://www.wavemetrics.com/
PatchMaster (Software), HEKA EPC‐10 (Hardware)	HEKA Electronic GmbH	https://www.heka.com/
ImageJ		https://imagej.nih.gov/ij/

### Methods and Protocols

#### Animals and postnatal injections

All experiments were done in compliance with national animal care guidelines and were approved by the University of Göttingen Board for Animal Welfare and the Animal Welfare Office of the State of Lower Saxony (permit number: 17‐2394). Postnatal AAV injections were made into scala tympani of the right ear through the round window (Akil *et al*, 2012; Jung *et al*, 2015). P5–7 WT C57Bl/6 mice were used for the injection of AAV9 virus under human synapsin promoter (pAAV9.*hSyn*.iGluSnFR.WPRE.SV40, Cat#98929‐AAV9, Addgene, USA, or produced in our own laboratory, see Reagents and Tools Table, and Huet and Rankovic, 2021) to drive transgenic expression of iGluSnFR in SGNs. In brief, the right ear was accessed through a dorsal incision. Once the round window membrane was located, a quartz capillary pipette was used to gently puncture it and inject ∼ 1–1.5 μl of pAAV9.*hSyn*.iGluSnFR (titer ≥ 1 × 10^13^ vg/ml). Subsequently the wound was sutured. The whole procedure was performed under general isoflurane anesthesia. For analgesia, buprenorphine (0.1 mg/kg) was injected subcutaneously prior the surgery, additional local analgesia (xylocaine) was applied to the skin and post‐surgery pain was covered by metamizol (1.33 mg/kg) provided in the drinking water for 5 days. The recovery of the animals was monitored on a daily basis. All animals were kept in a 12‐h light/dark cycle, with access to food and water *ad libitum* and together with the mother until the end of the weaning period (∼ P21). Injected WT mice were used for experiments either 1 week (P15–19) or 2 weeks (P21–26) after the injection.

#### Auditory brainstem recordings

Recordings of auditory brainstem responses (ABR) were performed on P29 mice as previously described (Strenzke *et al*, 2016). Briefly, mice were anesthetized with a combination of ketamine (125 mg/kg) and xylazine (2.5 mg/kg) intraperitoneally. The core temperature was maintained constant at 37°C using a heat blanket (Hugo Sachs Elektronik–Harvard Apparatus). The TDT II system run by BioSig software (Tucker Davis Technologies) or by MATLAB (MathWorks) was used for stimulus generation, presentation, and data acquisition. Tone bursts (6/12/24 kHz, 10‐ms plateau, 1 ms cos^2^ rise/fall) or clicks of 0.03 ms were presented at 40 Hz (tone bursts) or 20 Hz (clicks) in the free field ipsilaterally using a JBL 2402 speaker.

#### Immunohistochemistry and confocal microscopy

The cochlea was fixed in formaldehyde (4% in phosphate‐buffered saline [PBS], 1 h on ice). For immunostaining of the whole‐mount organs of Corti, the apical turns of organs of Corti were dissected out and washed three times with PBS. For immunostaining of the cryosections, the cochlea was decalcified in 0.5 M EDTA overnight. After a PBS washing step, the cochleae were incubated in 25% sucrose in PBS at 4°C. The cochlea was frozen in Tissue‐Tek and cryosectioned with section thickness of 16 µm.

The samples (cochlear apical turns or cryosections) were blocked with a goat serum dilution buffer (16% normal goat serum, 450 mM NaCl, 0.3% Triton X‐100, 20 mM phosphate buffer, pH 7.4) for 1 h at room temperature in a wet chamber. The blocking was followed by an overnight incubation with the primary antibodies at 4°C. After 3 × 5 min PBS washing steps, the samples were incubated with the secondary antibodies for 1 h at room temperature. Following the final 4 × 5 min PBS washing steps, the samples were mounted in mounting medium (Mowiol 4‐88, Sigma). The primary antibodies used were the following: mouse anti‐CtBP2 (1:200, BD Biosciences, 612044, See Reagents and Tools table)—to detect synaptic ribbons—chicken anti‐GFP (1:200, Abcam, 13970)—to detect iGluSnFR— and guinea pig anti‐parvalbumin (1:200, Synaptic Systems, 195004)—to detect SGNs, OHCs, and IHCs. Secondary goat antibodies were used with 1:200 dilution: Alexa Fluor 488‐conjugated anti‐chicken (Dianova, 703‐45‐155), Alexa Fluor 633‐conjugated anti‐mouse (Invitrogen, A31571), Alexa Fluor 488 anti‐chicken (Invitrogen, A11039), and Alexa Fluor 568 anti‐guinea pig (Invitrogen, A11075). Images were acquired using an Abberior Instruments Expert Line STED microscope, with excitation lasers at 488, 561, and 640 nm using a 1.4 NA 100× or 20× oil immersion objective, in confocal mode. Z‐step sizes of 0.5 or 0.6 µm were used with 100× or 20× objectives, respectively. Images were adjusted for brightness and contrast using ImageJ for illustration purposes.

#### Patch‐clamp recordings

The apical 2/3 turn of organs of Corti was acutely dissected from P15 to P26 animals in HEPES Hanks' solution containing (in mM): 5.36 KCl, 141.7 NaCl, 10 HEPES, 0.5 MgSO_4_, 1 MgCl_2_, 5.6 d‐glucose, and 3.4 l‐glutamine (pH 7.2, ∼ 300 mOsm/l). The IHC basolateral membranes were exposed by cleaning of nearby cells with a suction pipette by approaching from either pillar or modiolar side. All experiments were conducted at room temperature (20–25°C). Patch pipettes were made from GB150‐8P or GB150F‐8P borosilicate glass capillaries (Science Products, Hofheim, Germany) for perforated and ruptured patch‐clamp recordings, respectively. To decrease the capacitive noise, pipettes were coated with Sylgard and their tips were polished with a custom‐made microforge. All patch‐clamp recordings were done simultaneously with fluorescent imaging of iGluSnFR or of iGluSnFR and Ca^2+^.

##### Perforated patch recordings

Perforated patch‐clamp was performed as described previously (Moser & Beutner, 2000). For Ca^2+^ current and membrane capacitance (C_m_) measurements, the extracellular solution contained the following (in mM): 110 NaCl, 35 TEA‐Cl, 2.8 KCl, 1 MgCl_2_, 1 CsCl, 10 HEPES, 1.3 CaCl_2_, and 11.1 d‐glucose (pH 7.2, ∼ 305 mOsm/l) and was introduced into the recording chamber via a perfusion system. The pipette solution contained (in mM): 130 Cs‐gluconate, 10 TEA‐Cl, 10 4‐AP, 10 HEPES, 1 MgCl_2_, as well as 300 mg/ml amphotericin B (pH 7.17, ∼ 290 mOsm/l). The intracellular solution also contained the TAMRA‐conjugated dimeric CtBP2/RIBEYE‐binding dimer peptide (10 μM, Biosynthan, Germany, See Reagents and Tools table) (Francis *et al*, 2011; Wong *et al*, 2014). To label the synaptic ribbons, the peptide was introduced to the cell by rupturing the membrane patch toward the end of the recordings. The intracellular exposure to amphotericin (used to perforate the membrane for electrical access) did not result in a noticeable increase in IHC conductance and compromise IHC health. All the measurements were done via EPC‐10 amplifiers controlled by Patchmaster software (HEKA Elektronik, Germany). The holding potential was −87 for all the recordings. All voltages were corrected for liquid junction potential offline (17 mV). Currents were leak‐corrected using a p/10 protocol. Recordings were used only if the leak current was lower than 30 pA and the series resistance (Rs) was lower than 30 mOhm. The Lindau‐Neher technique was used to measure the C_m_ changes (Lindau & Neher, 1988). Exocytosis was quantified from C_m_ changes as described previously (Moser & Beutner, 2000; Neef *et al*, 2014). IHCs were stimulated by step depolarizations of different durations (2–100 ms, applied in a pseudo‐randomized manner) to −23 mV at intervals of 60–100 s (Fig EV1D–G). To probe voltage dependence of release, IHCs were step depolarized for 10 ms to potentials ranging from −62 to −22 mV in 5 mV increments in a pseudo‐randomized order (Figs 1 and EV2). For the Zn^2+^ perfusion experiments, 1 mM Zn^2+^ was slowly perfused in and out of the recording chamber, while IHCs were step depolarized for 10 ms to −23 mV simultaneously up to 20 times (Figs EV1A and B, and EV3D‐F).

##### Ruptured patch recordings

Ruptured patch experiments were performed in extracellular solution containing (in mM): 2.8 KCl, 102 NaCl, 10 HEPES, 1 CsCl_2_, 1 MgCl_2_, 35 TEA‐Cl, 2 mg/ml d‐Glucose, and 5 CaCl_2_ (pH 7.2, 300 mOsm). The patch pipette solution contained (in mM): 111 l‐glutamate, 1 MgCl_2_, 1 CaCl_2_, 10 EGTA, 13 TEA‐Cl, 20 HEPES, 4 Mg‐ATP, 0.3 Na‐GTP and 1 l‐Glutathione (pH 7.3, ∼ 290 mOsm). For fluorescent imaging, 800 μM of the low‐affinity chemical Ca^2+^ indicator Rhod‐FF tripotassium salt (Kd:19 μM, AAT Bioquest, USA, See Reagents and Tools table) was added to the intracellular solution. The recordings were discarded when the leak current exceeded −50 pA at −87 mV or R_S_ was greater than 15 MΩ within 4 min after break‐in. For Ca^2+^ imaging experiments, a voltage ramp (from −87 to +63 mV during 150 ms; 1 mV/ms) was applied to evoke Ca^2+^ influx (Figs 2 and 3).

For all IV recordings, the IHCs were step depolarized for 20 ms from −87 to +63 mV in 5 mV increments. The IV recordings were used to assess the fitness of the cell, and recordings were discarded when the Ca^2+^ current rundown exceeded 25%.

#### Spinning disk confocal imaging of Ca^2+^ and iGluSnFR

Imaging experiments were performed with a spinning disk confocal scanner (CSU22, Yokogawa, Germany) mounted on an upright microscope (Axio Examiner, Zeiss, Germany) with 63×, 1.0 NA objective (W Plan‐Apochromat, Zeiss). The spinning disk speed was set to 2,000 rpm to avoid uneven illumination. A scientific CMOS camera (Neo, Andor, Northern Ireland) with a pixel size of 103 nm was used to acquire images. iGluSnFR and Rhod‐FF or TAMRA peptide were excited by diode‐pumped solid‐state lasers with 491 nm and 561 nm wavelength, respectively (Cobolt AB).

##### iGluSnFR imaging

To avoid photobleaching, iGluSnFR‐expressing SGN boutons were detected via low intensity 491 nm excitation. The imaging plane for the target IHC was selected when several transduced boutons were visible, in the mid‐basal section of the cell, avoiding the high synapse density at the basal pole. A brief step depolarization was applied to the cell to check for the functional signal in the given plane. iGluSnFR fluorescence was acquired at 50 Hz simultaneously with patch‐clamp recordings. The iGluSnFR signal was evoked by step depolarizations of different durations to different voltage values, as it is specified in every dataset.

##### Sequential dual‐color imaging of Ca^2+^ and iGluSnFR

For the sequential dual‐color imaging of Ca^2+^ and glutamate release, as described above for iGluSnFR imaging, the imaging plane was selected based on the baseline fluorescence of iGluSnFR. Once the middle plane was set, the fluorescence of Rhod‐FF was imaged at 100 Hz while Ca^2+^ currents were triggered by applying five voltage ramps (from −87 to +63 mV, 1 mV/ms) in five alternating planes separated by 0.5 μm. To precisely control the Z‐plane, a piezo positioner for the objective (Piezosystem, Germany) was used. After the Ca^2+^ imaging, the iGluSnFR signal was acquired at 50 Hz by applying 50‐ms‐long step depolarizations from the holding potential of −87 to different voltage values. Depolarizations (to −57, −49, −45, −41, −37, −33, −25, −17 mV) were applied in a pseudo‐randomized manner and covered the dynamic range of IHC glutamate release.

#### Data analysis

##### Patch‐clamp recordings

Electrophysiological recordings were analyzed using custom‐written programs in Igor Pro 6.3. Whole‐cell Ca^2+^ charge (Q_Ca_) was calculated by the time integral of the leak‐subtracted current during the depolarization step. ∆C_m_ was calculated as the difference between the average C_m_ 400 ms before and after the depolarization, skipping the initial 100 ms after the depolarization.

##### Imaging of iGluSnFR

Image and further data analysis and visualization were done in Python (Python software foundation) with custom written code using the following Python libraries: NumPy, Pandas, Matplotlib, Skimage, SciPy, Sklearn, statmodels, and Seaborn.

###### Region of interest detection

The ∆F image was created by subtracting baseline fluorescence (F_0_, an average of 15 frames before stimulus) from the fluorescence images acquired during/after stimulation (F, an average of five frames). The ∆F image was median‐filtered with a two‐dimensional pixel array size of 4–6 depending on the signal amplitude. To create a mask for ROI detection, maximum entropy thresholding was applied to the median‐filtered ∆F image. To label and separate individual ROIs, a watershed segmentation algorithm was used. A single mask was generated per cell, using the recording with strongest stimulation, and applied for all images. Individual ROIs, corresponding to postsynaptic SGN boutons, were further confirmed by the nearby presence of presynaptic ribbon peptide (TAMRA‐conjugated dimeric CtBP2‐binding peptide). The fluorescence of every pixel in the defined ROI was averaged over time for further analysis. The background fluorescence was calculated by averaging 60 × 60 pixels in the pillar region of the image, where no iGluSnFR fluorescence is expected: By their anatomy, SGNs innervate IHCs and leave the cochlea toward the modiolus.

###### Analysis of fluorescence traces

The average background value was subtracted from the raw fluorescence traces (F). Following background subtraction, ∆F traces were generated by subtraction of mean baseline fluorescence (F_0_). ∆F was normalized to F_0_ to create ∆F/F_0_ traces. For peak detection, ∆F/F_0_ traces were smoothened using a Hanning window function with a window size of 7. To correct for photobleaching, we fitted a single exponential to ∆F/F_0_ traces. The area under the curve (AUC) was estimated by calculating the area between ∆F/F_0_ and the fit in an interval of 40 frames from the beginning of the stimulus.

###### Estimation of sensitivity of iGluSnFR and ∆C_m_

For iGluSnFR, mean of 10 frames before stimulation is compared pairwise per synapse with the mean of four frames after stimulation. For ∆C_m_, mean of 400 points before and after stimulation is used for pairwise comparison per cell.

###### Estimation of the time to peak and decay constant

To obtain decay constant (*τ*_off_), the following function was fitted to the 30 points of photobleaching corrected ∆F/F_0_ traces after stimulation.

$$\Delta F/F_{0}=A*\left(1‐e^{‐t\;/\;\tau_{\mathrm{on}}}\right)*e^{‐t\;/\;\tau_{\mathrm{off}}}$$

$$\mathrm{time}\mathrm{to}\mathrm{peak}\left(\mathrm{ms}\right)=\tau_{\mathrm{on}}*\log\left(\tau_{\mathrm{off}}\;/\;\tau_{\mathrm{on}}+1\right)$$

###### Estimation of the RRP size and time constant of RRP depletion

To assess the dynamics of RRP and sustained exocytosis, we fitted a sum of an exponential and linear function (Pangrsic *et al*, 2015) to ∆Cm and iGluSnFR‐AUC for different stimulus durations.

$$\Delta C_{m}\left(t\right)or{\mathrm{AUC}}_{\mathrm{iGluSnFR}}\left(t\right)=\mathrm{RRPsize}*{\left(1‐e^{t\;/\;\tau }\right)}^{n}+\mathrm{slope}*t.$$

With the assumptions of ∆C_m_ of ~ 40 aF contributed by a single SV (Grabner & Moser, 2018) and ~ 12 AZs (Meyer *et al*, 2009) for the apical IHCs, we obtained iGluSnFR‐AUC increase of 0.23 a.u. per SV.

##### Sequential imaging of Ca^2+^ and iGluSnFR

###### ROI detection–iGluSnFR

The ROIs were picked as described above. Differently, a Gaussian filter with sigma of 1–3 was applied consistent with the detection of Ca^2+^ hot spots (see below). A mean mask was generated per cell using all the recordings. ROIs were confirmed by the presence of a corresponding Ca^2+^ “hot spot”.

###### ROI detection–Rhod‐FF

Similarly, a ∆F image was created from the mean time series, in this case, by averaging all the trials from five recorded planes. This ∆F image was treated the same way as described for iGluSnFR‐ROI detection. The created mask was applied to all recording planes and the plane with the maximum ∆F for a given ROI was used for further analysis. This way we used the plane with the highest signal for each Ca^2+^ “hot spot”.

###### Analysis of fluorescence traces

To remove the noise caused by the spinning disk speed at 2,000 rpm (33.3 Hz), obvious in the Fourier amplitude spectrum, the raw traces were filtered with a 33.3 Hz band‐stop filter. The obtained traces were background‐subtracted and normalized to F_0_ as described above. Fluorescence–voltage (FV) relations for iGluSnFR were estimated from the step depolarizations to different voltage values. iGluSnFR‐AUC for each depolarization was calculated as described above.

###### Estimation of the parameters of threshold, dynamic range, and V_1/2_

A Boltzmann fit was used to estimate the two fitting parameters: voltage of half‐maximum activation (V_1/2_) and slope factor (*k*) of glutamate release.

$${\mathrm{AUC}}_{\mathrm{iGluSnFR}}\left(V\right)=\frac{1}{1+e^{\frac{V_{1/2}‐V}{k}}}.$$

For Ca^2+^ imaging, FV curves were estimated from voltage ramps. To optimize the raw FV traces against noise such as readout or shot noise from the CCD camera, the following equation was used (Ohn *et al*, 2016):

$$F_{\mathrm{Rhod}‐\mathrm{FF}}\left(V\right)=F_{0}+\frac{g_{\max}\left(V‐V_{r}\right)}{1+e^{\frac{V_{1/2}‐V}{k}}}.$$

The slope factor (*k*) was obtained with this equation. The resulting fit was used to estimate V_1/2_ by minimizing the scalar at the mid‐point. The reversal potential (V_r_) was fixed to +47.6 mV after LJ potential (17 mV) correction. In addition, this fit was used to calculate the fractional activation curve, dividing it by the extrapolated linear fit to the decay of fluorescence. To estimate fractional V_1/2,Pactivation_ and *k*_1/2,Pactivation_ (see Fig EV6), an additional Boltzmann fitting was done.

The peak of the Rhod‐FF signal was obtained by averaging three frames corresponding to the voltage values between −17 and 3 mV during ramp depolarization. Dynamic ranges were calculated from the fits as the voltage range of 10–90% of the maximal activation. Threshold was calculated as the voltage value where there is 10% of the maximal activation. Note that V_1/ 2_ values obtained from fluorescence traces after denoising (band‐stop filter at 33.3 Hz) were comparable to the ones from the raw fluorescence traces.

###### Estimating the Ca^2+^ cooperativity

FV fits for Rhod‐FF or Q_Ca_ and iGluSnFR‐AUC were plotted against each other in the voltage range of −57 to −17 mV. To obtain single‐synapse glutamate release–Ca^2+^ signal/whole‐cell Ca^2+^ charge relationship, a power function was fitted:

$${\mathrm{AUC}}_{\mathrm{iGluSnFR}}\left(V\right)=A{\left(F_{\mathrm{Rhod}‐\mathrm{FF}}\right)}^{m}$$

$${\mathrm{AUC}}_{\mathrm{iGluSnFR}}\left(V\right)=A{\left(Q_{\mathrm{Ca}}\right)}^{m}.$$

###### Calculation of the position of the synapse along cell's pillar–modiolar axis

To estimate the cell boundary, an ellipse was fitted to the baseline fluorescence of Rhod‐FF. We defined the pillar–modiolar axis of the cell as the major axis of the ellipse. We calculated the shortest distance from the center of a given Ca^2+^ "hot spot" to the normalized major axis. A number was assigned to a given synapse on the normalized scale from 0 (pillar side) to 1 (modiolar side).

###### *K*‐mean clustering and principal components analysis

*K*‐means clustering algorithm (*K* = 3) was applied to the whole dataset of 11 synaptic properties for three clusters (see Fig 5). Principal component analysis was used to display the clusters obtained by the *K*‐means clustering.

#### Statistical analysis

All the statistical tests were performed in Python (Python Software Foundation). Averages are expressed as mean ± SD or as mean ± SEM (specified in the figure legends), and box plots indicate 25–75 quartile with whiskers reaching from 10 to 90%. Datasets were checked for normal distribution by D'Agostino & Pearson omnibus normality test and for equality of variances. For normally distributed data, unpaired two‐tailed student's *t*‐test was applied, and for non‐normally distributed data, Mann–Whitney *U*‐test was used. Dependent samples were compared by paired *t*‐test (for normally distributed data) or Wilcoxon's signed rank test (for non‐normally distributed data). Comparison of dispersion was performed by Levene's test. We used one‐way ANOVA for multiple comparisons followed by *post hoc* Tukey's test. The Pearson correlation coefficient was used to test for linear correlation. Significant differences are reported as *P ≤ 0.05, **P ≤ 0.01, ***P ≤ 0.001.

## Author contributions

TM and ÖDÖ designed the study. ÖDÖ performed the experiments and the analysis. TM and ÖDÖ prepared the manuscript.

## Conflict of interests

The authors declare that they have no conflict of interest.

## Supporting information



Appendix

Expanded View Figures PDF

Review Process File

## Data Availability

This study includes no data deposited in external repositories.

## References

[embj2020106010-bib-0400] Akil O , Seal RP , Burke K , Wang C , Alemi A , During M , Edwards RH , Lustig LR (2012) Restoration of hearing in the VGLUT3 knockout mouse using virally mediated gene therapy. Neuron 75: 283–293 22841313 10.1016/j.neuron.2012.05.019 PMC3408581

[embj2020106010-bib-0001] Armbruster M , Dulla CG , Diamond JS (2020) Effects of fluorescent glutamate indicators on neurotransmitter diffusion and uptake. eLife 9: e54441 32352378 10.7554/eLife.54441 PMC7255799

[embj2020106010-bib-0002] Ashmore J (2008) Cochlear outer hair cell motility. Physiol Rev 88: 173–210 18195086 10.1152/physrev.00044.2006

[embj2020106010-bib-0003] Beutner D , Voets T , Neher E , Moser T (2001) Calcium dependence of exocytosis and endocytosis at the cochlear inner hair cell afferent synapse. Neuron 29: 681–690 11301027 10.1016/s0896-6273(01)00243-4

[embj2020106010-bib-0004] Böhme MA , Grasskamp AT , Walter AM (2018) Regulation of synaptic release‐site Ca^2+^ channel coupling as a mechanism to control release probability and short‐term plasticity. FEBS Lett 592: 3516–3531 29993122 10.1002/1873-3468.13188

[embj2020106010-bib-0005] Brandt A , Khimich D , Moser T (2005) Few CaV1. 3 channels regulate the exocytosis of a synaptic vesicle at the hair cell ribbon synapse. J Neurosci 25: 11577–11585 16354915 10.1523/JNEUROSCI.3411-05.2005 PMC6726013

[embj2020106010-bib-0006] Charizopoulou N , Lelli A , Schraders M , Ray K , Hildebrand MS , Ramesh A , Srisailapathy CRS , Oostrik J , Admiraal RJC , Neely HR *et al* (2011) Gipc3 mutations associated with audiogenic seizures and sensorineural hearing loss in mouse and human. Nat Commun 2: 201 21326233 10.1038/ncomms1200 PMC3105340

[embj2020106010-bib-0007] Druckenbrod NR , Hale EB , Olukoya OO , Shatzer WE , Goodrich LV (2020) Neuronal processes and glial precursors form a scaffold for wiring the developing mouse cochlea. Nat Commun 11: 5866 33203842 10.1038/s41467-020-19521-2 PMC7672226

[embj2020106010-bib-0008] Dulon D , Safieddine S , Jones SM , Petit C (2009) Otoferlin is critical for a highly sensitive and linear calcium‐dependent exocytosis at vestibular hair cell ribbon synapses. J Neurosci 29: 10474–10487 19710301 10.1523/JNEUROSCI.1009-09.2009 PMC2966717

[embj2020106010-bib-0009] Eggermann E , Bucurenciu I , Goswami SP , Jonas P (2012) Nanodomain coupling between Ca^2+^ channels and sensors of exocytosis at fast mammalian synapses. Nat Rev Neurosci 13: 7–21 10.1038/nrn3125 PMC3617475 22183436

[embj2020106010-bib-0401] Francis AA , Mehta B , Zenisek D (2011) Development of new peptide‐based tools for studying synaptic ribbon function. J Neurophysiol 106: 1028–1037 21653726 10.1152/jn.00255.2011 PMC3154815

[embj2020106010-bib-0010] Frank T , Khimich D , Neef A , Moser T (2009) Mechanisms contributing to synaptic Ca^2+^ signals and their heterogeneity in hair cells. Proc Natl Acad Sci USA 106: 4483–4488 19246382 10.1073/pnas.0813213106 PMC2657422

[embj2020106010-bib-0011] Furman AC , Kujawa SG , Liberman MC (2013) Noise‐induced cochlear neuropathy is selective for fibers with low spontaneous rates. J Neurophysiol 110: 577–586 23596328 10.1152/jn.00164.2013 PMC3742994

[embj2020106010-bib-0012] Goldberg JM , Fernandez C (1971) Physiology of peripheral neurons innervating semicircular canals of the squirrel monkey. I. Resting discharge and response to constant angular accelerations. J Neurophysiol 34: 635–660 5000362 10.1152/jn.1971.34.4.635

[embj2020106010-bib-0013] Goutman JD , Glowatzki E (2007) Time course and calcium dependence of transmitter release at a single ribbon synapse. Proc Natl Acad Sci USA 104: 16341–16346 17911259 10.1073/pnas.0705756104 PMC2042208

[embj2020106010-bib-0404] Grabner CP , Moser T (2018) Individual synaptic vesicles mediate stimulated exocytosis from cochlear inner hair cells. Proc Natl Acad Sci USA 115: 12811–12816 30463957 10.1073/pnas.1811814115 PMC6294930

[embj2020106010-bib-0014] Graydon CW , Cho S , Li G‐L , Kachar B , von Gersdorff H (2011) Sharp Ca^2+^ nanodomains beneath the ribbon promote highly synchronous multivesicular release at hair cell synapses. J Neurosci 31: 16637–16650 22090491 10.1523/JNEUROSCI.1866-11.2011 PMC3235473

[embj2020106010-bib-0015] Griesinger CB , Richards CD , Ashmore JF (2005) Fast vesicle replenishment allows indefatigable signalling at the first auditory synapse. Nature 435: 212–215 15829919 10.1038/nature03567

[embj2020106010-bib-0016] Han Y , Kaeser PS , Südhof TC , Schneggenburger R (2011) RIM determines Ca^2+^ channel density and vesicle docking at the presynaptic active zone. Neuron 69: 304–316 21262468 10.1016/j.neuron.2010.12.014 PMC3259453

[embj2020106010-bib-0017] Heil P , Neubauer H (2010) Summing across different active zones can explain the quasi‐linear Ca^2+^‐dependencies of exocytosis by receptor cells. Front Synaptic Neurosci 2: 148 21423534 10.3389/fnsyn.2010.00148 PMC3059696

[embj2020106010-bib-0018] Heil P , Peterson AJ (2015) Basic response properties of auditory nerve fibers: a review. Cell Tissue Res 361: 129–158 25920587 10.1007/s00441-015-2177-9

[embj2020106010-bib-0405] Huet AT , Rankovic V (2021) Application of targeting‐optimized chronos for stimulation of the auditory pathway. Methods Mol Biol 2191: 261–285 32865750 10.1007/978-1-0716-0830-2_16

[embj2020106010-bib-0019] Jean P , Özçete ÖD , Tarchini B , Moser T (2019) Intrinsic planar polarity mechanisms influence the position‐dependent regulation of synapse properties in inner hair cells. Proc Natl Acad Sci USA 116: 9084–9093 30975754 10.1073/pnas.1818358116 PMC6500111

[embj2020106010-bib-0020] Johnson SL , Beurg M , Marcotti W , Fettiplace R (2011) Prestin‐driven cochlear amplification is not limited by the outer hair cell membrane time constant. Neuron 70: 1143–1154 21689600 10.1016/j.neuron.2011.04.024 PMC3143834

[embj2020106010-bib-0021] Johnson SL , Olt J , Cho S , von Gersdorff H , Marcotti W (2017) The coupling between Ca^2+^ channels and the exocytotic Ca^2+^ sensor at hair cell ribbon synapses varies tonotopically along the mature cochlea. J Neurosci 37: 2471–2484 28154149 10.1523/JNEUROSCI.2867-16.2017 PMC5354352

[embj2020106010-bib-0022] Jung S , Oshima‐Takago T , Chakrabarti R , Wong AB , Jing Z , Yamanbaeva G , Picher MM , Wojcik SM , Göttfert F , Predoehl F *et al* (2015) Rab3‐interacting molecules 2α and 2β promote the abundance of voltage‐gated CaV1.3 Ca^2+^ channels at hair cell active zones. Proc Natl Acad Sci USA 112: E3141–E3149 26034270 10.1073/pnas.1417207112 PMC4475996

[embj2020106010-bib-0023] Kandel ER , Schwartz JH , Jessell T (2012) Principles of neural science, New York, NY: McGraw‐Hill Medical

[embj2020106010-bib-0024] Kantardzhieva AV , Liberman MC , Sewell WF (2013) Quantitative analysis of ribbons, vesicles, and cisterns at the cat inner hair cell synapse: correlations with spontaneous rate. J Comp Neurol 521: 3260–3271 23787810 10.1002/cne.23345 PMC4309284

[embj2020106010-bib-0025] Kiang NYS , Watanabe T , Thomas EC , Clark LF (1965) Discharge patterns of single fibers in the cat’s auditory nerve Cambridge. Cambridge, MA: MIT Press

[embj2020106010-bib-0026] Krinner S , Butola T , Jung S , Wichmann C , Moser T (2017) RIM‐binding protein 2 promotes a large number of CaV1.3 Ca^2+^‐channels and contributes to fast synaptic vesicle replenishment at hair cell active zones. Front Cell Neurosci 11: 334 29163046 10.3389/fncel.2017.00334 PMC5673845

[embj2020106010-bib-0027] Liberman MC (1978) Auditory‐nerve response from cats raised in a low‐noise chamber. J Acoust Soc Am 63: 442–455 670542 10.1121/1.381736

[embj2020106010-bib-0028] Liberman MC (1980) Morphological differences among radial afferent fibers in the cat cochlea: an electron‐microscopic study of serial sections. Hear Res 3: 45–63 7400048 10.1016/0378-5955(80)90007-6

[embj2020106010-bib-0029] Liberman M (1982) Single‐neuron labeling in the cat auditory nerve. Science 216: 1239–1241 7079757 10.1126/science.7079757

[embj2020106010-bib-0030] Liberman LD , Wang H , Liberman MC (2011) Opposing gradients of ribbon size and ampa receptor expression underlie sensitivity differences among cochlear‐nerve/hair‐cell synapses. J Neurosci 31: 801–808 21248103 10.1523/JNEUROSCI.3389-10.2011 PMC3290333

[embj2020106010-bib-0031] Liberman LD , Liberman MC (2016) Postnatal maturation of auditory‐nerve heterogeneity, as seen in spatial gradients of synapse morphology in the inner hair cell area. Hear Res 339: 12–22 27288592 10.1016/j.heares.2016.06.002 PMC5018435

[embj2020106010-bib-0402] Lindau M , Neher E (1988) Patch‐clamp techniques for time‐resolved capacitance measurements in single cells. Pflügers Archiv Eur J Phy 411: 137–146 3357753 10.1007/BF00582306

[embj2020106010-bib-0032] Liu KSY , Siebert M , Mertel S , Knoche E , Wegener S , Wichmann C , Matkovic T , Muhammad K , Depner H , Mettke C *et al* (2011) RIM‐binding protein, a central part of the active zone, is essential for neurotransmitter release. Science 334: 1565–1569 22174254 10.1126/science.1212991

[embj2020106010-bib-0033] Marvin JS , Borghuis BG , Tian L , Cichon J , Harnett MT , Akerboom J , Gordus A , Renninger SL , Chen T‐W , Bargmann CI *et al* (2013) An optimized fluorescent probe for visualizing glutamate neurotransmission. Nat Methods 10: 162–170 23314171 10.1038/nmeth.2333 PMC4469972

[embj2020106010-bib-0034] Merchan‐Perez A , Liberman MC (1996) Ultrastructural differences among afferent synapses on cochlear hair cells: correlations with spontaneous discharge rate. J Comp Neurol 371: 208–221 8835727 10.1002/(SICI)1096-9861(19960722)371:2<208::AID-CNE2>3.0.CO;2-6

[embj2020106010-bib-0035] Meyer AC , Frank T , Khimich D , Hoch G , Riedel D , Chapochnikov NM , Yarin YM , Harke B , Hell SW , Egner A *et al* (2009) Tuning of synapse number, structure and function in the cochlea. Nat Neurosci 12: 444–453 19270686 10.1038/nn.2293

[embj2020106010-bib-0036] Michanski S , Smaluch K , Steyer AM , Chakrabarti R , Setz C , Oestreicher D , Fischer C , Möbius W , Moser T , Vogl C *et al* (2019) Mapping developmental maturation of inner hair cell ribbon synapses in the apical mouse cochlea. Proc Natl Acad Sci USA 116: 6415–6424 30867284 10.1073/pnas.1812029116 PMC6442603

[embj2020106010-bib-0037] Moser T , Beutner D (2000) Kinetics of exocytosis and endocytosis at the cochlear inner hair cell afferent synapse of the mouse. Proc Natl Acad Sci USA 97: 883–888 10639174 10.1073/pnas.97.2.883 PMC15425

[embj2020106010-bib-0038] Moser T , Neef A , Khimich D (2006) Mechanisms underlying the temporal precision of sound coding at the inner hair cell ribbon synapse. J Physiol 576: 55–62 16901948 10.1113/jphysiol.2006.114835 PMC1995636

[embj2020106010-bib-0039] Moser T , Grabner CP , Schmitz F (2019) Sensory processing at ribbon synapses in the retina and the cochlea. Physiol Rev 100: 103–144 31373863 10.1152/physrev.00026.2018

[embj2020106010-bib-0040] Nakamura Y , Harada H , Kamasawa N , Matsui K , Rothman JS , Shigemoto R , Silver RA , DiGregorio DA , Takahashi T (2015) Nanoscale distribution of presynaptic Ca^2+^ channels and its impact on vesicular release during development. Neuron 85: 145–158 25533484 10.1016/j.neuron.2014.11.019 PMC4305191

[embj2020106010-bib-0406] Neef J , Jung S , Wong AB , Reuter K , Pangrsic T, Chakrabarti R , Kugler S , Lenz C , Nouvian R , Boumil RM et al (2014) Modes and regulation of endocytic membrane retrieval in mouse auditory hair cells. J Neurosci 34: 705–716 24431429 10.1523/JNEUROSCI.3313-13.2014 PMC3891952

[embj2020106010-bib-0041] Neef J , Urban NT , Ohn T‐L , Frank T , Jean P , Hell SW , Willig KI , Moser T (2018) Quantitative optical nanophysiology of Ca^2+^ signaling at inner hair cell active zones. Nat Commun 9: 290 29348575 10.1038/s41467-017-02612-y PMC5773603

[embj2020106010-bib-0042] Odermatt B , Nikolaev A , Lagnado L (2012) Encoding of luminance and contrast by linear and nonlinear synapses in the retina. Neuron 73: 758–773 22365549 10.1016/j.neuron.2011.12.023 PMC3314971

[embj2020106010-bib-0043] Oheim M , van't Hoff M , Feltz A , Zamaleeva A , Mallet J‐M , Collot M (2014) New red‐fluorescent calcium indicators for optogenetics, photoactivation and multi‐color imaging. Biochim Biophys Acta BBA Mol Cell Res 1843: 2284–2306 10.1016/j.bbamcr.2014.03.010 24681159

[embj2020106010-bib-0044] Ohn T‐L , Rutherford MA , Jing Z , Jung S , Duque‐Afonso CJ , Hoch G , Picher MM , Scharinger A , Strenzke N , Moser T (2016) Hair cells use active zones with different voltage dependence of Ca^2+^ influx to decompose sounds into complementary neural codes. Proc Natl Acad Sci USA 113: E4716–E4725 27462107 10.1073/pnas.1605737113 PMC4987782

[embj2020106010-bib-0045] Pangrsic T , Gabrielaitis M , Michanski S , Schwaller B , Wolf F , Strenzke N , Moser T (2015) EF‐hand protein Ca^2+^ buffers regulate Ca^2+^ influx and exocytosis in sensory hair cells. Proc Natl Acad Sci USA 112: E1028–E1037 25691754 10.1073/pnas.1416424112 PMC4352837

[embj2020106010-bib-0046] Petitpré C , Wu H , Sharma A , Tokarska A , Fontanet P , Wang Y , Helmbacher F , Yackle K , Silberberg G , Hadjab S *et al* (2018) Neuronal heterogeneity and stereotyped connectivity in the auditory afferent system. Nat Commun 9: 3691 30209249 10.1038/s41467-018-06033-3 PMC6135759

[embj2020106010-bib-0047] Rebola N , Reva M , Kirizs T , Szoboszlay M , Lőrincz A , Moneron G , Nusser Z , DiGregorio DA (2019) Distinct nanoscale calcium channel and synaptic vesicle topographies contribute to the diversity of synaptic function. Neuron 104: 693–710 31558350 10.1016/j.neuron.2019.08.014

[embj2020106010-bib-0048] Roux I , Safieddine S , Nouvian R , Grati M , Simmler M‐C , Bahloul A , Perfettini I , Le Gall M , Rostaing P , Hamard G *et al* (2006) Otoferlin, defective in a human deafness form, is essential for exocytosis at the auditory ribbon synapse. Cell 127: 277–289 17055430 10.1016/j.cell.2006.08.040

[embj2020106010-bib-0049] Ruel J , Nouvian R , D’Aldin CG , Pujol R , Eybalin M , Puel J‐L (2001) Dopamine inhibition of auditory nerve activity in the adult mammalian cochlea. Eur J Neurosci 14: 977–986 11595036 10.1046/j.0953-816x.2001.01721.x

[embj2020106010-bib-0050] Russell IJ , Sellick PM (1983) Low‐frequency characteristics of intracellularly recorded receptor potentials in guinea‐pig cochlear hair cells. J Physiol 338: 179–206 6875955 10.1113/jphysiol.1983.sp014668 PMC1197189

[embj2020106010-bib-0051] Sachs MB , Abbas PJ (1974) Rate versus level functions for auditory‐nerve fibers in cats: tone‐burst stimuli. J Acoust Soc Am 56: 1835–1847 4443483 10.1121/1.1903521

[embj2020106010-bib-0052] Shrestha BR , Chia C , Wu L , Kujawa SG , Liberman MC , Goodrich LV (2018) Sensory neuron diversity in the inner ear is shaped by activity. Cell 174: 1229–1246 30078709 10.1016/j.cell.2018.07.007 PMC6150604

[embj2020106010-bib-0053] Song Q , Shen P , Li X , Shi L , Liu L , Wang J , Yu Z , Stephen K , Aiken S , Yin S *et al* (2016) Coding deficits in hidden hearing loss induced by noise: the nature and impacts. Sci Rep 6: 1–13 27117978 10.1038/srep25200 PMC4846864

[embj2020106010-bib-0500] Strenzke N , Chakrabarti R , Al‐Moyed H , Müller A , Hoch G , Pangrsic T , Yamanbaeva G , Lenz C , Pan K‐T , Auge E et al (2016) Hair cell synaptic dysfunction, auditory fatigue and thermal sensitivity in otoferlin Ile515Thr mutants. EMBO J 35: e201694564 10.15252/embj.201694564 PMC5283603 27729456

[embj2020106010-bib-0054] Sun S , Babola T , Pregernig G , So KS , Nguyen M , Su S‐SM , Palermo AT , Bergles DE , Burns JC , Müller U (2018) Hair cell mechanotransduction regulates spontaneous activity and spiral ganglion subtype specification in the auditory system. Cell 174: 1247–1263 30078710 10.1016/j.cell.2018.07.008 PMC6429032

[embj2020106010-bib-0055] Taberner AM , Liberman MC (2005) Response properties of single auditory nerve fibers in the mouse. J Neurophysiol 93: 557–569 15456804 10.1152/jn.00574.2004

[embj2020106010-bib-0056] Vincent PFY , Bouleau Y , Safieddine S , Petit C , Dulon D (2014) Exocytotic machineries of vestibular type I and cochlear ribbon synapses display similar intrinsic otoferlin‐dependent Ca^2+^ sensitivity but a different coupling to Ca^2+^ channels. J Neurosci Off J Soc Neurosci 34: 10853–10869 10.1523/JNEUROSCI.0947-14.2014 PMC6705247 25122888

[embj2020106010-bib-0057] Vyleta NP , Jonas P (2014) Loose coupling between Ca^2+^ channels and release sensors at a plastic hippocampal synapse. Science 343: 665–670 24503854 10.1126/science.1244811

[embj2020106010-bib-0058] Wen B , Wang GI , Dean I , Delgutte B (2009) Dynamic range adaptation to sound level statistics in the auditory nerve. J Neurosci 29: 13797–13808 19889991 10.1523/JNEUROSCI.5610-08.2009 PMC2774902

[embj2020106010-bib-0303] Winegar BD , Lansman JB (1990) Voltage‐dependent block by zinc of single calcium channels in mouse myotubes. J Physiol 425: 563–578 2170633 10.1113/jphysiol.1990.sp018118 PMC1189863

[embj2020106010-bib-0059] Winter IM , Robertson D , Yates GK (1990) Diversity of characteristic frequency rate‐intensity functions in guinea pig auditory nerve fibres. Hear Res 45: 191–202 2358413 10.1016/0378-5955(90)90120-e

[embj2020106010-bib-0060] Wong AB , Jing Z , Rutherford MA , Frank T , Strenzke N , Moser T (2013) Concurrent maturation of inner hair cell synaptic Ca^2+^ influx and auditory nerve spontaneous activity around hearing onset in mice. J Neurosci 33: 10661–10666 23804089 10.1523/JNEUROSCI.1215-13.2013 PMC6618488

[embj2020106010-bib-0061] Wong AB , Rutherford MA , Gabrielaitis M , Pangršič T , Göttfert F , Frank T , Michanski S , Hell S , Wolf F , Wichmann C *et al* (2014) Developmental refinement of hair cell synapses tightens the coupling of Ca^2+^ influx to exocytosis. EMBO J 33: 247–264 24442635 10.1002/embj.201387110 PMC3989618

[embj2020106010-bib-0062] Zampini V , Johnson SL , Franz C , Lawrence ND , Münkner S , Engel J , Knipper M , Magistretti J , Masetto S , Marcotti W (2010) Elementary properties of Ca _V_ 1.3 Ca^2+^ channels expressed in mouse cochlear inner hair cells: biophysics of Ca^2+^ channels in IHCs. J Physiol 588: 187–199 19917569 10.1113/jphysiol.2009.181917 PMC2817446

[embj2020106010-bib-0201] Zenisek D , Horst NK , Merrifield C , Sterling P , Matthews G (2004) Visualizing synaptic ribbons in the living cell. J Neurosci 24: 9752–9759 15525760 10.1523/JNEUROSCI.2886-04.2004 PMC6730242

[embj2020106010-bib-0063] Zhang L , Engler S , Koepcke L , Steenken F , Köppl C (2018) Concurrent gradients of ribbon volume and AMPA‐receptor patch volume in cochlear afferent synapses on gerbil inner hair cells. Hear Res 364: 81–89 29631778 10.1016/j.heares.2018.03.028

