# Supplementary material for: A sensory cell diversifies its output by varying Ca2+ influx‐release coupling among active zones
Source: EMBO J. 2020 Dec 21;40(5):e106010. doi: 10.15252/embj.2020106010 (PMC7917556; doi:10.15252/embj.2020106010)
Supplement: Supplementary file 1 — Appendix [file EMBJ-40-e106010-s001.pdf]

Appendix - Table of Contents

Appendix Figure S1. AAV9.*hSyn*.iGluSnFR virus shows a high transduction efficiency in SGNs throughout the cochlea. Related to Fig 1.

Appendix Figure S2. Custom Python pipeline for iGluSnFR ROI detection. Related to Fig 1.

Appendix Figure S3. Effects of the imaging plane selection on the peak iGluSnFR amplitude. Related to Fig 1.

Appendix Figure S4. Mild rundown of the functional iGluSnFR signal upon repetitive stimulation. Related to Fig 1.

Appendix Figure S5. iGluSnFR shows a significant rise in response to 2 ms step depolarizations, while a significant change can only be detected by ΔC_m in response to 5 ms step depolarizations. Related to Figure EV1.

Appendix Figure S6. iGluSnFR does not show an obvious sign of saturation when probed by brief step depolarizations. Related to Fig EV1.

Appendix Figure S7. Voltage dependence of synaptic Ca^{2+} influx. Related to Fig 3.

Appendix Figure S8. Voltage dependence of synaptic glutamate release. Related to Fig 3.

Appendix Figure S9. Apparent Ca^{2+} dependence of synapses in individual IHCs. Related to Fig 3.

Appendix Figure S10. No significant correlation of the apparent Ca^{2+} dependence of release with the position along the pillar-modiolar axis. Related to Fig 4.

Appendix Figure S11. K-means clustering with K = 2 an K =4. Related to Fig 5.

Appendix Figure S12. Correlation between the synaptic properties and the principal components shown in Fig 5. Related to Fig 5.

Appendix Figure S13. The heterogeneity of Ca^{2+} dependence of release was preserved in the recorded ages (P21-26). Related to Fig 3.

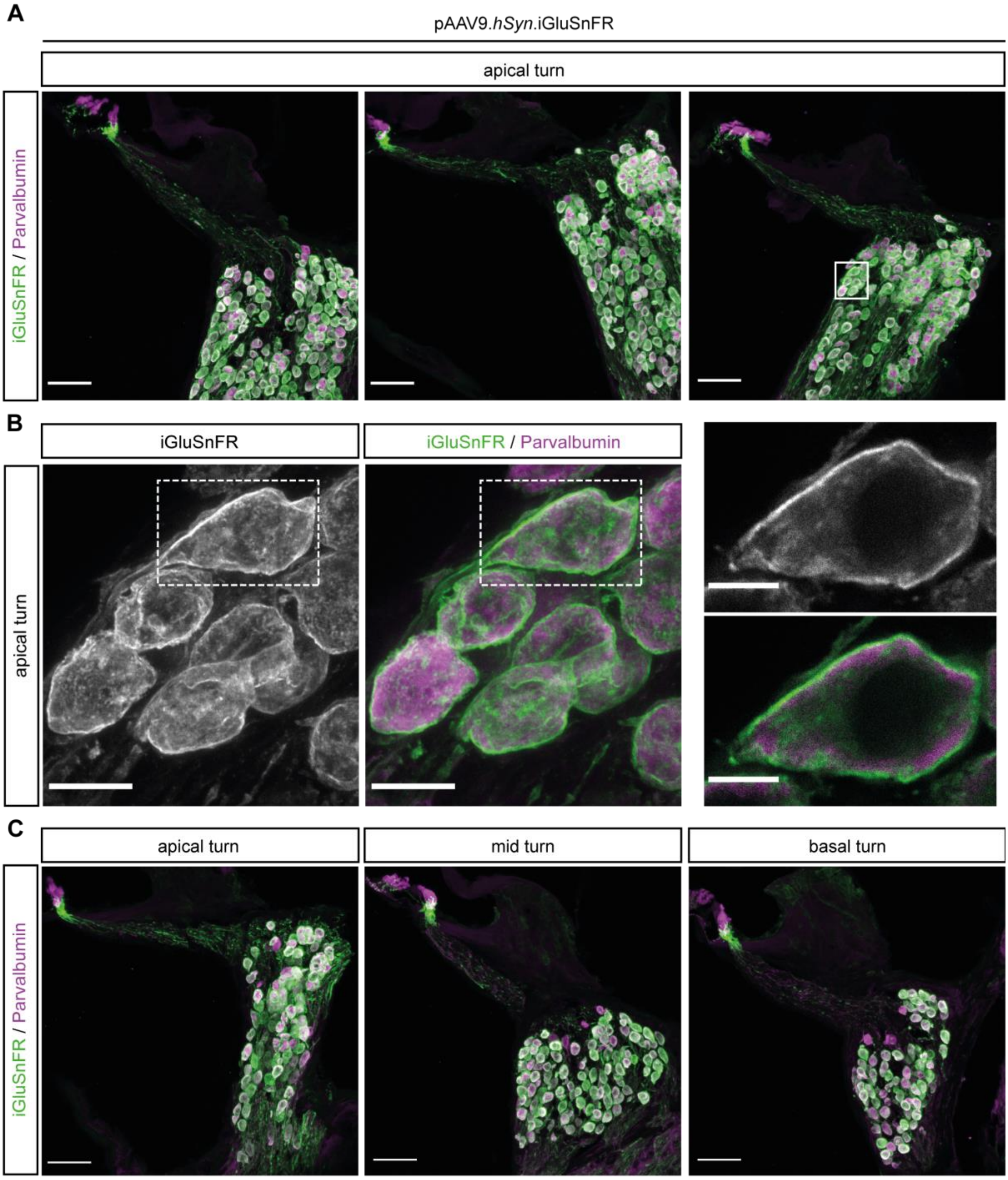

Appendix Figure S1. AAV9.*hSyn*.iGluSnFR virus shows a high transduction efficiency in SGNs throughout the cochlea. Related to Fig 1. Maximum intensity projections of mid-modiolar cochlear cryosections 3 weeks after the postnatal injection of the pAAV9.*hSyn*.iGluSnFR virus at P6, immunolabeled for iGluSnFR (GFP), and IHCs, OHCs, and SGNs (parvalbumin).

A. Exemplary sections of the apical turn. The mean transduction efficiency was 99.10 ± 1.46 % of the parvalbumin-expressing SGNs in the apical turn (4 cryosections from an injected cochlea).

B. A close-up image of the depicted region in right panel of A. Right panel shows the close-up single section image of the depicted region in the left and the middle panels. Note the little overlap of

iGluSnFR expression with parvalbumin, as parvalbumin is localized to the cytoplasm, while iGluSnFR is localized to the membrane.

C. Exemplary images of the apical, mid, and basal turn from a cochlear cryosection.

Scale bars (A and C): 50 µm, (B) 10 µm, (B, close up) 5 µm. Z-step size: 0.5 µm.

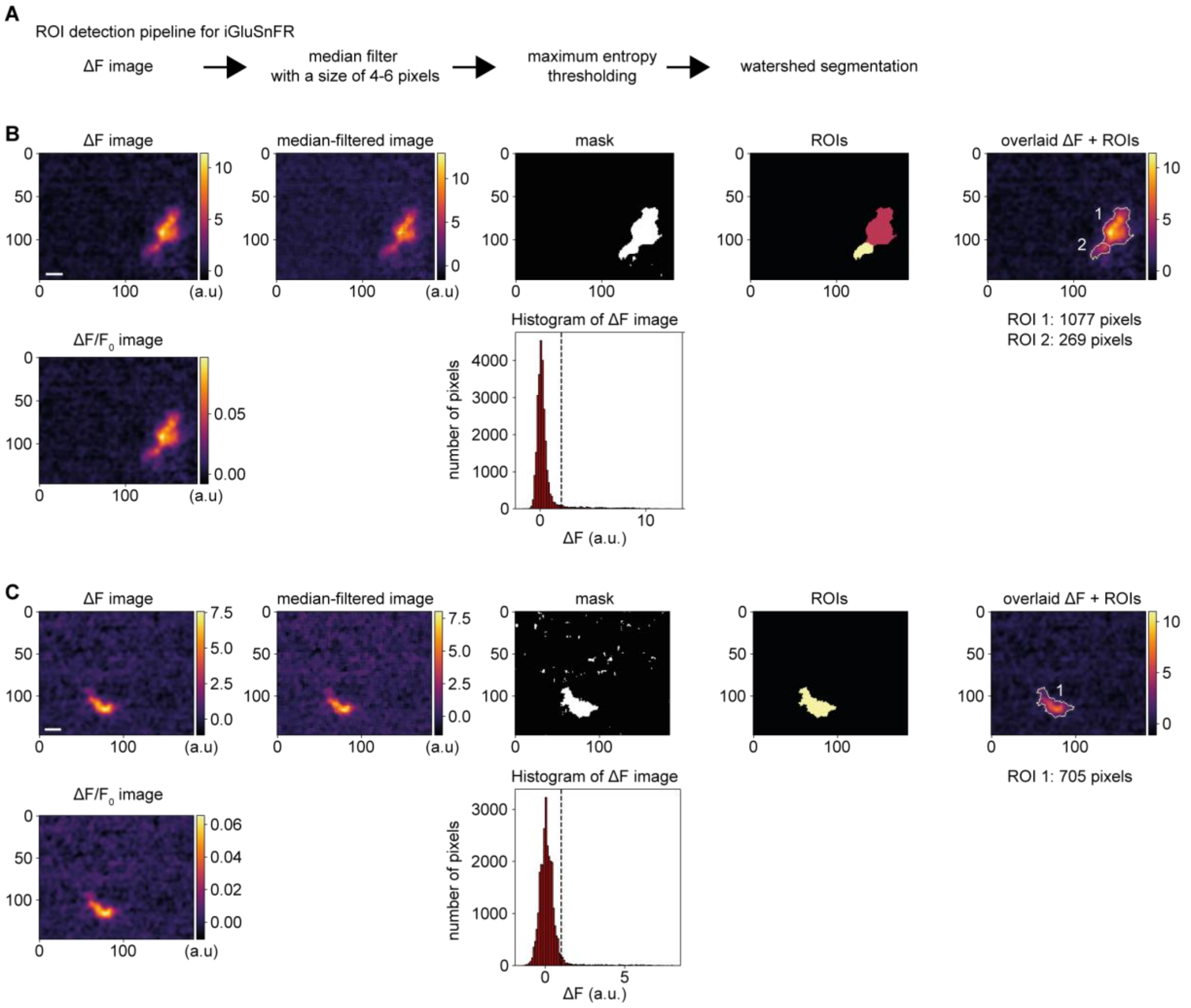

Appendix Figure S2. Custom Python pipeline for iGluSnFR ROI detection. Related to Fig 1.

A. To detect the iGluSnFR ROI, we sequentially create the ΔF image, and apply median-filter followed by the maximum entropy thresholding and watershed segmentation. The ΔF was created by subtracting baseline fluorescence (F_0, an average of 15 frames before stimulus) from the fluorescence images acquired during/after stimulation (F, an average of 5 frames). The ΔF image was median-filtered with an array sized 4-6 pixels depending on the signal amplitude. To create a mask for ROI detection, maximum entropy thresholding was applied to the median-filtered ΔF image. To label and separate individual ROIs, a watershed segmentation algorithm was used. A single mask was generated per cell, using the recording with strongest stimulation, and applied for all images. Individual ROIs, corresponding to postsynaptic SGN boutons, were further confirmed by the nearby presence of presynaptic ribbon peptide (TAMRA-conjugated dimeric CtBP2-binding peptide). The fluorescence of every pixel in the defined ROI was averaged over time for further analysis. The background fluorescence was calculated by averaging 60 × 60 pixels in the pillar region of the image, where no

iGluSnFR fluorescence is expected: By their anatomy, SGNs innervate IHCs and leave the cochlea towards the modiolus.

B-C. Pipeline applied on the images from Figure 1B, IHC 1 (B) and IHC 2 (C). Scale bars: 2 µm.

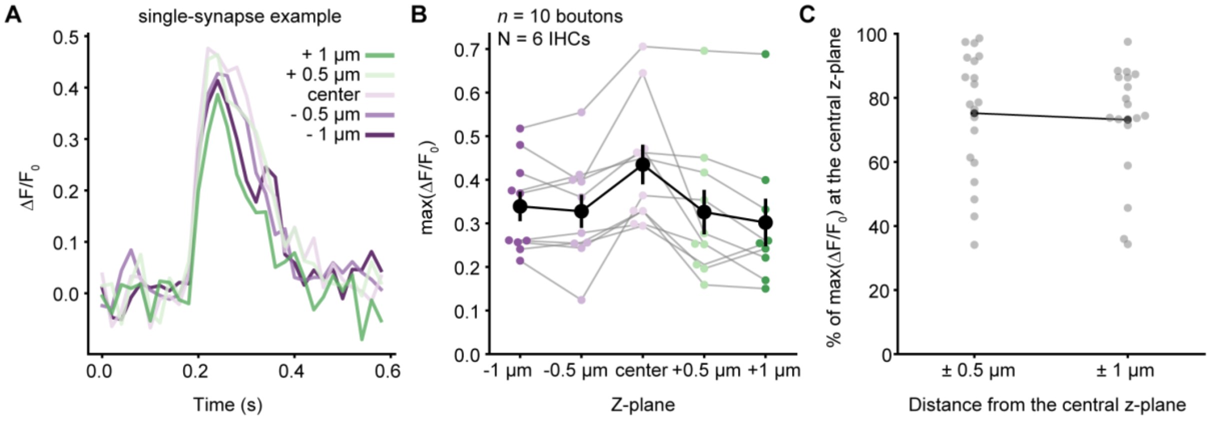

Appendix Figure S3. Effects of the imaging plane selection on the peak iGluSnFR amplitude. Related to Fig 1.

A. Exemplary single-synapse iGluSnFR signal in response to 50-ms-long step depolarizations applied in 5 different z-planes separated by 0.5 µm. The plane with the highest signal was determined as the center plane.

B. The peak of iGluSnFR signal plotted as a function of the imaging plane (mean ± SEM; ruptured patch-clamp, 10 mM intracellular EGTA, 5 mM $[Ca^{2+}]_e$; *n* = 10 boutons, N = 6 IHCs from 4 mice).

C. The percentage of the peak of iGluSnFR signal at the central z-plane compared to the signal in planes ±0.5 µm and ±1 µm from the center plane. The peak of iGluSnFR shows 24.79 ± 4.33% reduction for the planes ±0.5 µm from the center, and 26.83 ± 4.05 % reduction for the planes ±1 µm from the center.

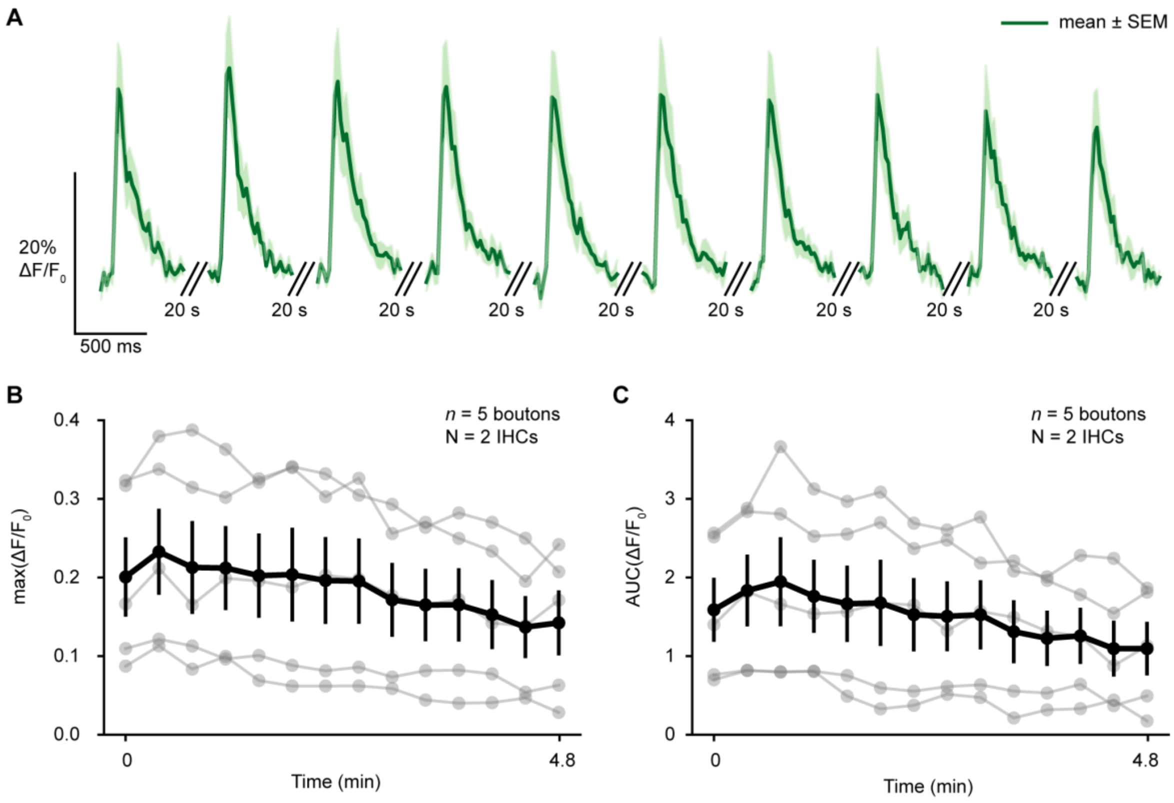

Appendix Figure S4. Mild rundown of the functional iGluSnFR signal upon repetitive stimulation. Related to Fig 1.

A. Average $\Delta F/F_0$ iGluSnFR traces in response to 20-ms-long step depolarizations from the holding potential of -87 mV to -17 mV applied in every 20 seconds over 5 mins. Recordings were done in organs of Corti of P26 WT mice injected with AAV9.*hSyn*.iGluSnFR virus (ruptured patch-clamp, 10 mM intracellular EGTA, 5 mM $[Ca^{2+}]_e$, *n* = 5 boutons, N = 2 IHCs from 2 mice). Shaded areas show ± SEM.

B-C. The change of the peak (B) and the AUC (C) of the iGluSnFR signal over time upon 14 repetitive 50-ms-long step depolarizations, applied as described in A. The mean peak amplitude (B) of the first three points showed 36.75 ± 7.03 % decrease compared to the mean of the last three points. The AUC amplitude (C) showed 39.21 ± 5.16 % decrease compared to the mean of the last three points. The bold lines indicate mean ± SEM.

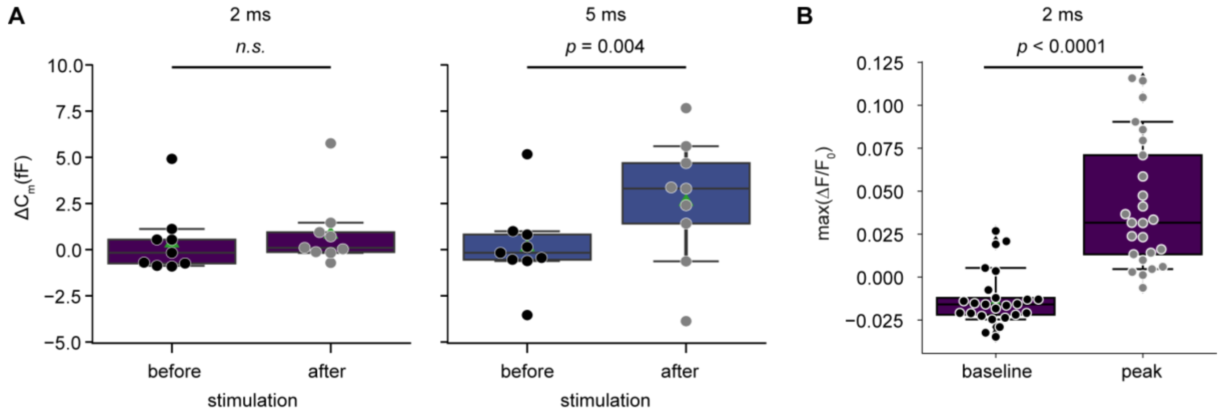

Appendix Figure S5. iGluSnFR shows a significant rise in response to 2 ms step depolarizations, while a significant change can only be detected by ΔC_m in response to 5 ms step depolarizations. Related to Figure EV1.

A ΔC_m before and after the stimulation (in response to 2 ms or 5 ms step depolarizations) calculated from the data shown in Fig EV1D. Mean of 400 points before and after stimulation is used for pairwise comparison per cell. ΔC_m were detectable only at 5 ms ($p = 0.004$, paired t-test, $N = 11$ IHCs).

B ΔF-iGluSnFR before and after the stimulation (only in response to 2 ms step depolazation is shown) simultaneously recorded with C_m, calculated from the data shown in Fig EV1D. Mean of 10 frames before stimulation is compared pairwise per synapse with the mean of four frames after stimulation. ΔF-iGluSnFR became significant already at 2 ms ($p < 0.0001$, Wilcoxon signed-rank test, $n = 31$ synapses from 11 IHCs).

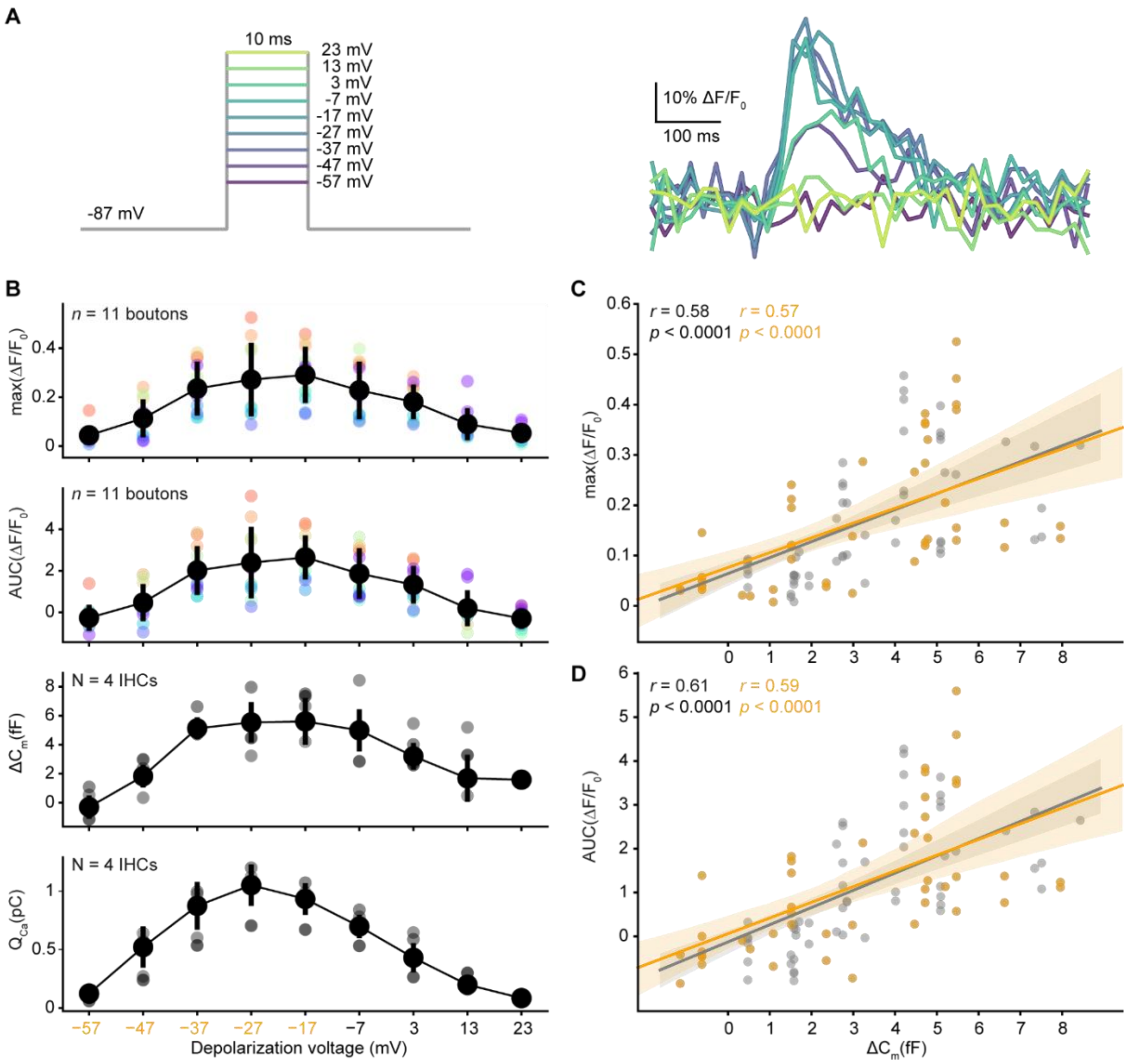

Appendix Figure S6. iGluSnFR does not show an obvious sign of saturation when probed by brief step depolarizations. Related to Fig EV1.

A. Exemplary single-synapse iGluSnFR signals (right) in response to 10-ms-long step depolarizations from the holding potential of -87 mV to -57 mV to 23 mV in 10 mV steps (applied in pseudo-randomized order) (left). Recordings were done in organs of Corti of P15-19 WT mice injected with AAV9.*hSyn*.iGluSnFR virus (perforated patch-clamp, 1.3 mM $[Ca^{2+}]_e$, *n* = 11 boutons, N = 4 IHCs from 4 mice).

B. The peak and the AUC of iGluSnFR signal, corresponding whole-cell ΔC_m and QCa plotted as a function of depolarization voltage (mean ± SD).

C-D. The relation of whole-cell ΔC_m and the peak (C) or the AUC (D) of the iGluSnFR signal. Orange data points indicate the negative voltage range. Both the peak and the AUC of iGluSnFR correlate with the whole-cell ΔC_m in the negative voltage range (from -57 mV to -17 mV) (Pearson's $r = 0.57$, $p < 0.0001$, Pearson's $r = 0.59$, $p < 0.0001$, respectively), as well in the whole range (both the gray and orange data points) (Pearson's $r = 0.58$, $p < 0.0001$, Pearson's $r = 0.61$, $p < 0.0001$, respectively). Linear regression analysis (solid lines) and the associated 95% confidence intervals (shaded area). Orange solid line

indicates the analysis of the data points from the negative voltage range, while gray one takes all the data points (both orange and gray) into consideration.

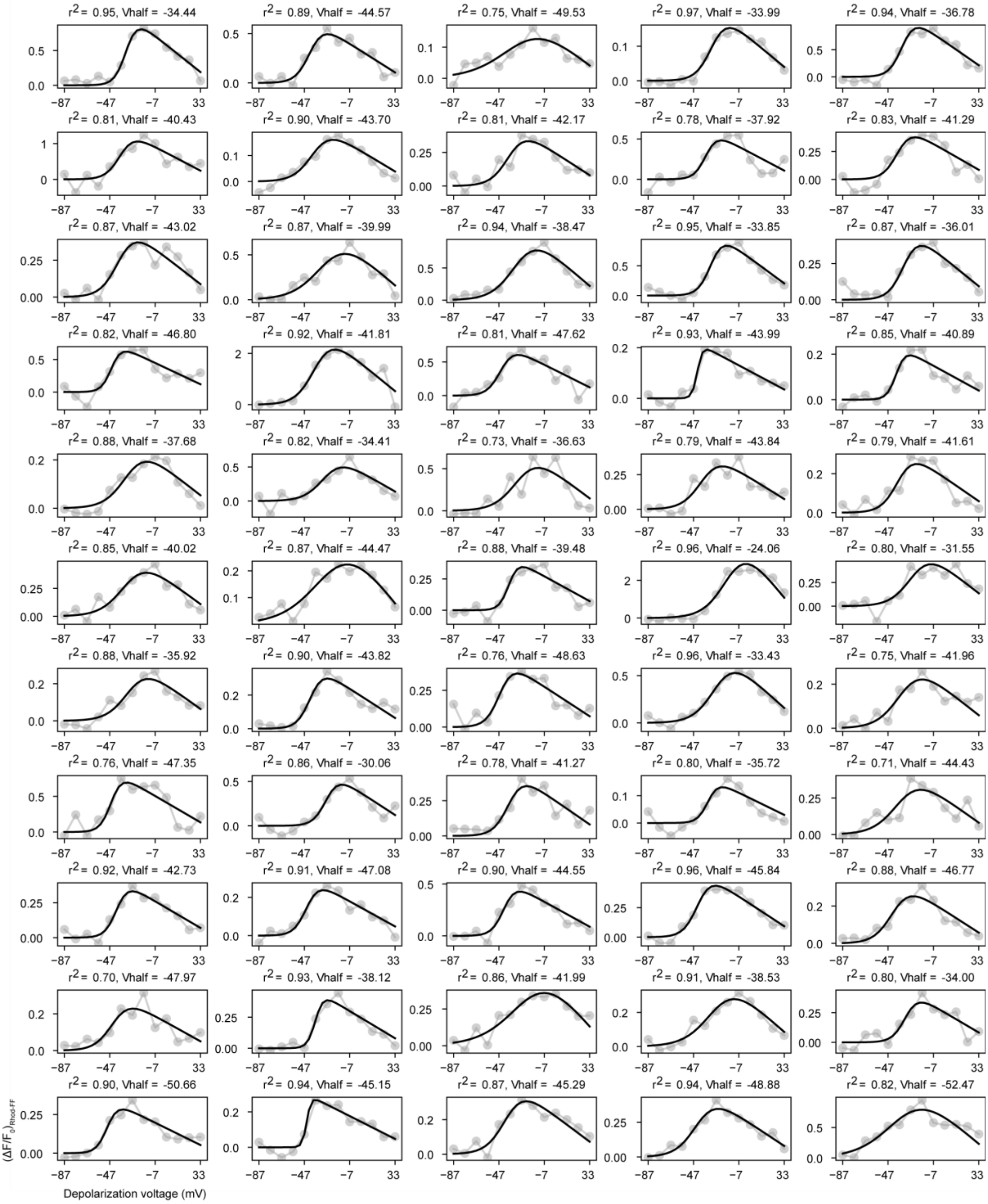

Appendix Figure S7. Voltage dependence of synaptic Ca^{2+} influx presented in Figure 3B. Related to Fig 3.

Individual $\Delta F/F_0$ of Rhod-FF fluorescence traces from Fig 3B in response to a voltage ramp and corresponding modified Boltzman fits (bold line) are depicted. The $V_{1/2}$ of synaptic Ca^{2+} influx was

calculated from the fits. Both the goodness of the fit (r^2) and $V_{1/2}$ of individual synapses are depicted on top of each trace (*n* = 55 synapses, N = 34 IHCs from 28 mice).

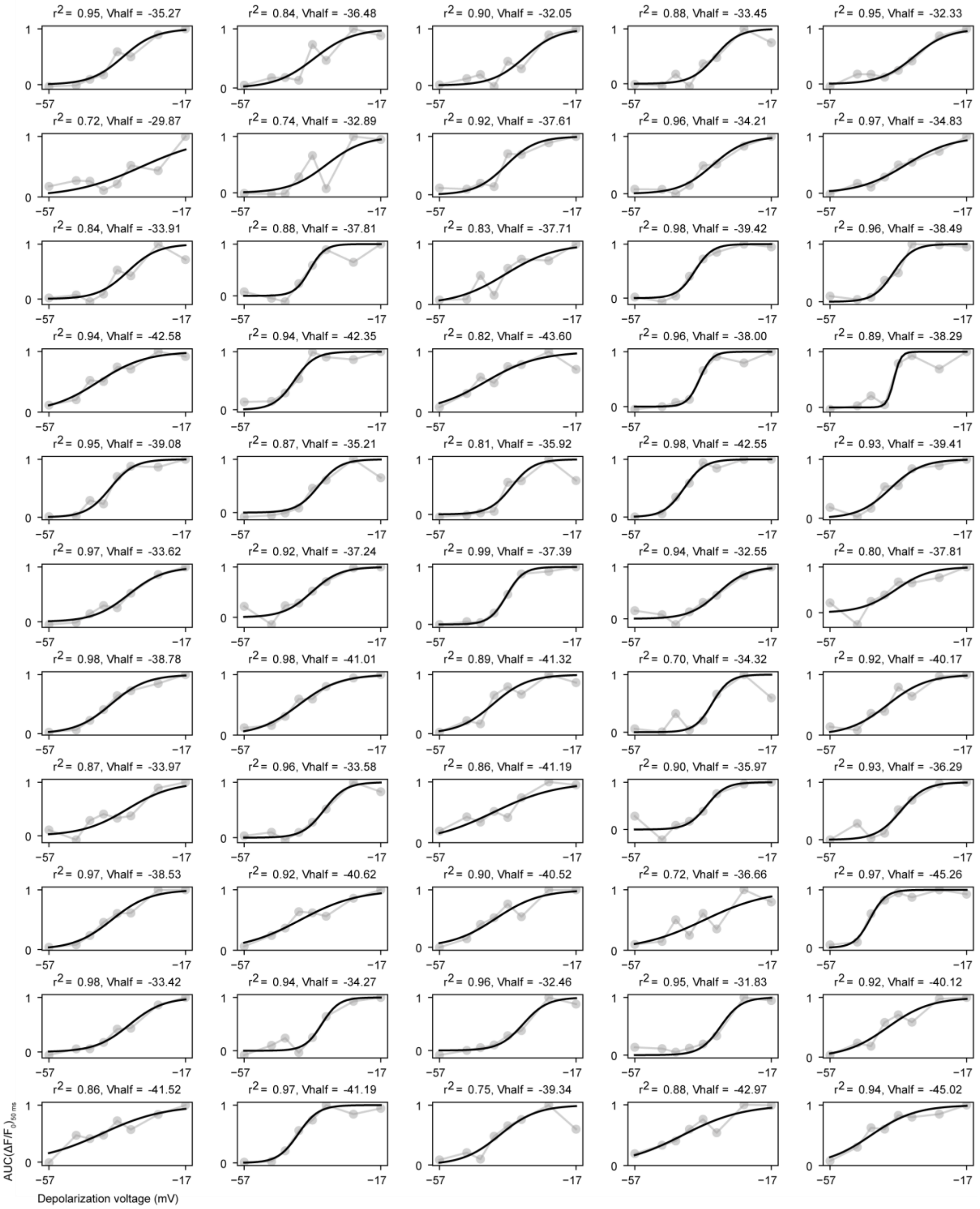

Appendix Figure S8. Voltage dependence of synaptic glutamate release presented in Figure 3C. Related to Fig 3.

Individual normalized iGluSnFR-AUC from Fig. 3C in response to 50-ms-step depolarizations to the given voltage values from the holding potential of -87 mV. The corresponding Boltzman fits (bold line) are depicted. The $V_{1/2}$ of synaptic glutamate was calculated from the fits. Both the goodness of the fit

(r^2) and $V_{1/2}$ of individual synapses are depicted on top of each trace (*n* = 55 synapses, N = 34 IHCs from 28 mice).

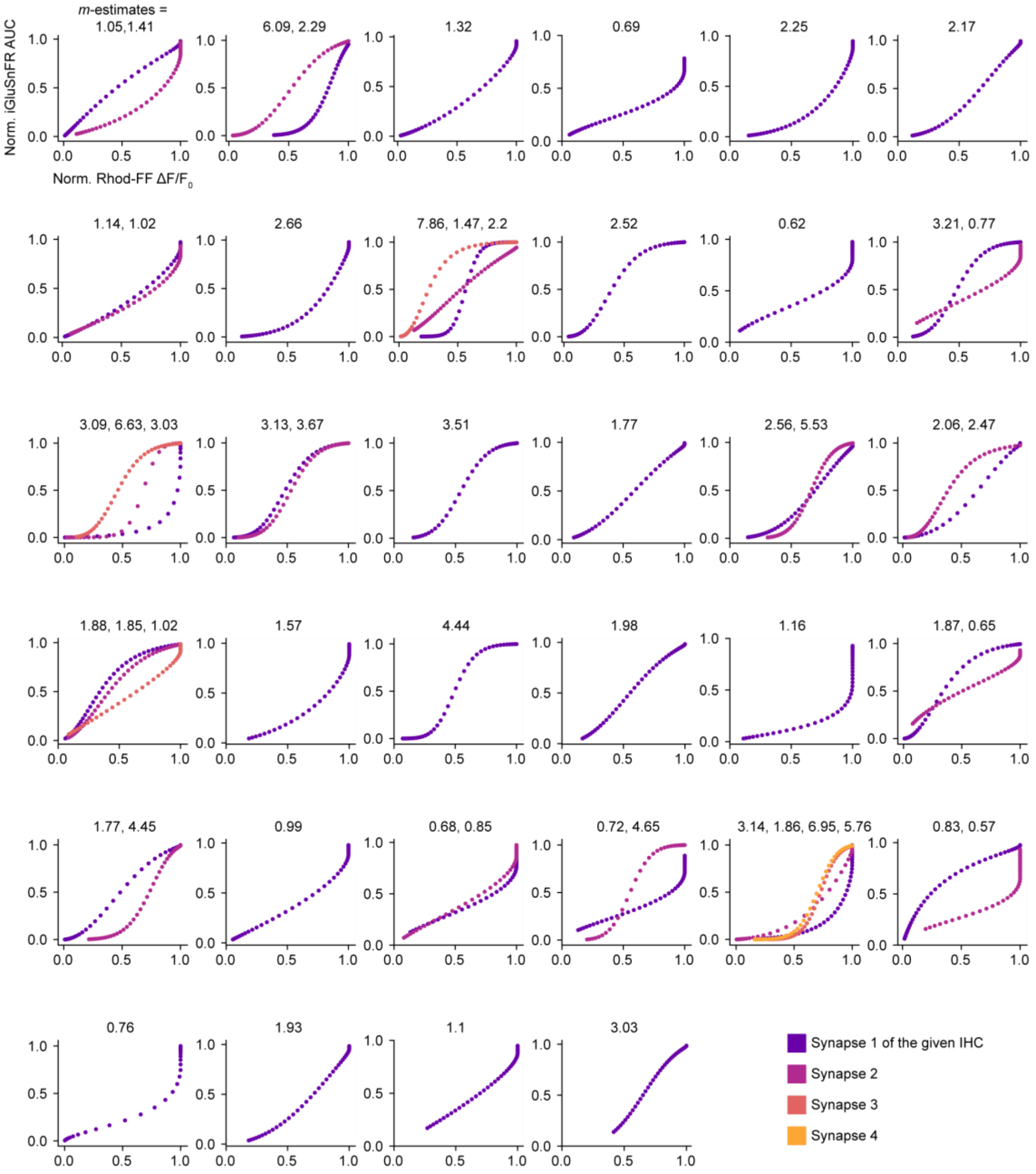

Appendix Figure S9. Apparent Ca^{2+} dependence of synapses in individual IHCs presented in Figure 3E. Related to Figure 3.

The relations of synaptic Ca^{2+} influx (normalized modified Boltzmann fit on $\Delta F/F_0$ of Rhod-FF) and glutamate release (normalized Boltzmann fit on iGluSnFR-AUC) were plotted per IHC. Ca^{2+} cooperativities (*m*-estimates) were obtained by individual power function fitting until 25% of normalized iGluSnFR response. Synapses of a given IHC are color coded and displayed on the lower right corner.

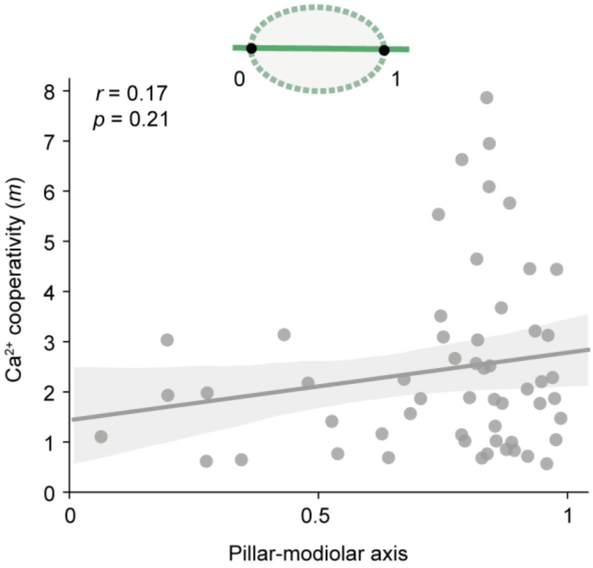

Appendix Figure S10. No significant correlation of the apparent Ca^{2+} dependence of release with the position along the pillar-modiolar axis.

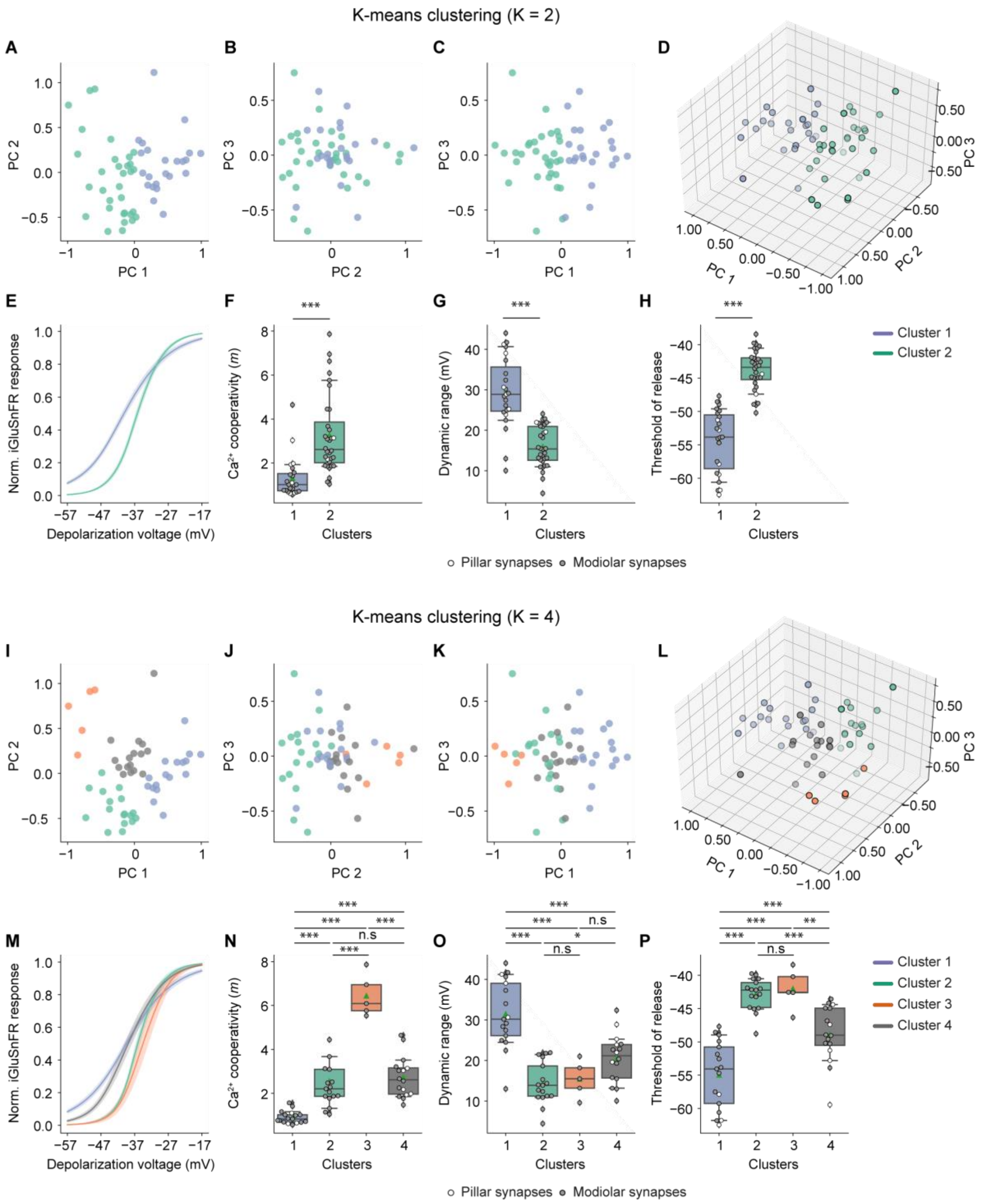

Appendix Figure S11. K-means clustering with K = 2 an K =4. Related to Fig 5. 11 synaptic properties (except for the positional and whole-cell information; see Fig 5) of 55 synapses (N = 34 IHCs from 28 mice, ruptured patch-clamp, 10 mM intracellular EGTA, 5 mM $[Ca^{2+}]_e$) were used for the PCA and K-means clustering.

A-C and I-K The 2D plots of the first three principal components (PCs), labeled based on the clusters obtained by K-means clustering algorithm (K=2 or K=4, respectively).

D and L 3D plot showing the first three PCs.

E and M Mean glutamate release (iGluSnFR-AUC) as a function of depolarization voltage in the identified clusters (mean ± SEM). The Ca^{2+}-cooperativity (*m*) (F, N), dynamic range (G, O), and glutamate release threshold (V_{10}) (H, P) show differences for the three clusters. Cluster 1 is composed of linear synapses with wider dynamic range and lower threshold compared to the other clusters. Pillar synapses are depicted as white-filled circles, and modiolar ones are depicted as gray-filled circles. The clusters were compared by Mann-Whitney U test (for 2 clusters) or by one-way ANOVA test, followed by a posthoc Tukey's test (for 4 clusters). * $p \leq 0.05$, ** $p \leq 0.01$, *** $p \leq 0.001$

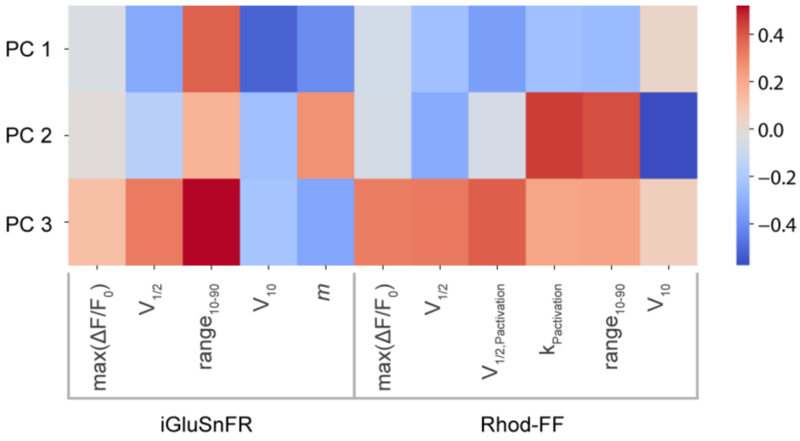

Appendix Figure S12. Correlation between the synaptic properties and the principal components shown in Figure 5. Related to Fig 5. PC 1 shows highest positive correlation (0.38) with the dynamic range of glutamate release, and highest negative correlation (-0.51) with the threshold of glutamate release. PC 2 shows the highest positive correlation (0.44) with slope factor of the fractional activation of Ca^{2+} channels, and highest negative correlation (-0.57) with threshold of synaptic Ca^{2+} influx. PC 3 shows the highest positive correlation (0.51) with dynamic range of glutamate release, and highest negative correlation (-0.33) with the Ca^{2+} cooperativity of release (*m*).

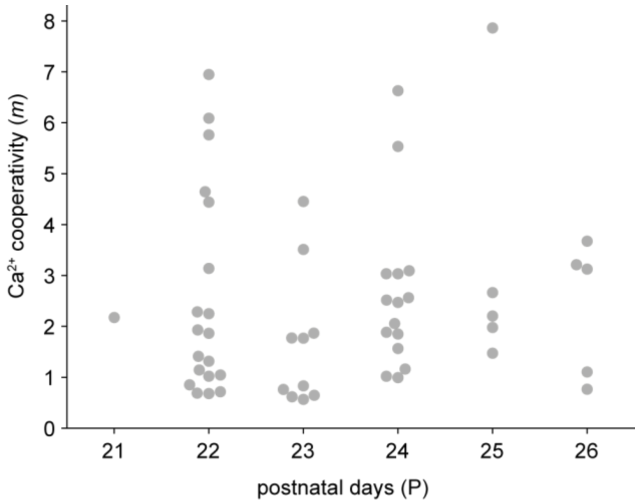

Appendix Figure S13. The heterogeneity of Ca^{2+} dependence of release was preserved in the recorded ages (P21-26). Related to Fig 3.
