# Supplementary material for: A sensory cell diversifies its output by varying Ca2+ influx‐release coupling among active zones
Source: EMBO J. 2020 Dec 21;40(5):e106010. doi: 10.15252/embj.2020106010 (PMC7917556; doi:10.15252/embj.2020106010)
Supplement: Supplementary file 2 — Expanded View Figures PDF [file EMBJ-40-e106010-s002.pdf]

Expanded View Figures

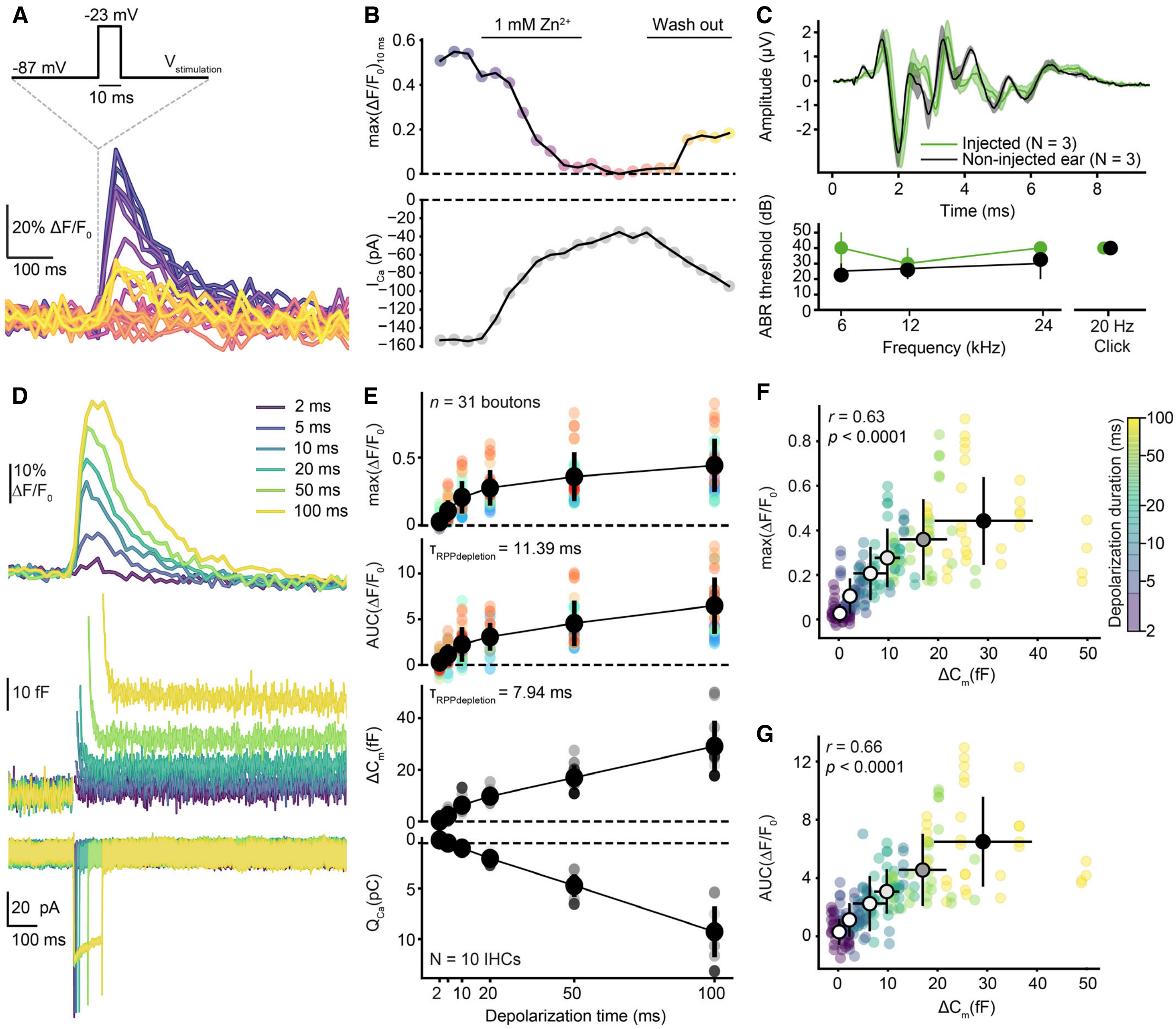

H

	$\tau_{RPPdepletion}$	Total RRP size	Sustained rate	Sustained rate per AZ (SVs/s)
**ΔCm(fF)**	7.94 ms	4.94 fF/s<br>123.5 SVs	242 fF/s	504 SVs/s
**AUC(ΔF/F$_0$)**	11.39 ms	2.41 a.u.	42.7 a.u./s	185 SVs/s

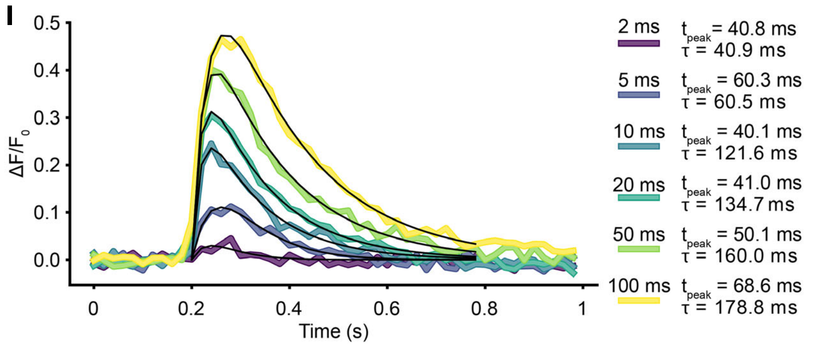

Figure EV1.

Figure EV1. Characterization and validation of iGluSnFR for reporting IHC exocytosis. Related to Fig 1.

A Exemplary single-synapse iGluSnFR signal in response to repetitive 10-ms-long step depolarizations to −23 mV from the holding potential (−87 mV) as Ca^{2+} channel blocker Zn^{2+} (1 mM) is perfused in and out of the recording chamber. The temporal sequence of the recordings is encoded by color; darker colors indicate earlier time points as in panel (B) (top).

B The time course of the peak iGluSnFR response (top; $max(\Delta F/F_0)_{10ms}$) from (A) and corresponding whole-cell peak Ca^{2+} current (bottom; perforated patch, 1.3 mM $[Ca^{2+}]_e$). The whole-cell Ca^{2+} influx decreases with the perfusion of Zn^{2+}.

C (top) ABR waveforms of P29 WT mice, injected with AAV9.*hSyn*.iGluSnFR virus at P6, were recorded in response to 80 dB clicks (mean ± SEM, three animals). The non-injected ear was used as a control. (bottom) ABR thresholds of the injected ear and the non-injected control were comparable. A statistical test was not applied due to the small sample size. The presence of iGluSnFR expression was confirmed by immunostainings after ABR recordings.

D–G iGluSnFR signal as a readout of glutamate release increases with stimulus duration along with the IHC's capacitance change (ΔC_m). (D) Average responses of iGluSnFR (top), whole-cell C_m (middle), and Ca^{2+} currents (bottom) upon step depolarizations to −23 mV from the holding potential of −87 mV for durations from 2 to 100 ms (color coded). Recordings were done in organs of Corti of P15–19 WT mice injected with AAV9.*hSyn*.iGluSnFR virus (perforated patch-clamp, 1.3 mM $[Ca^{2+}]_e$, $n = 31$ boutons, $N = 10$ IHCs from eight mice). (E) The peak and the AUC of iGluSnFR signal, corresponding whole-cell ΔC_m, and Q_{Ca} plotted as a function of depolarization duration (mean ± SD). (F, G) The relation of whole-cell ΔC_m and the peak (F) or the AUC (G) of the iGluSnFR signal (mean ± SD, $n = 31$ boutons, $N = 10$ IHCs from 8 mice). Both the peak and the AUC of iGluSnFR response correlate with the whole-cell ΔC_m (Pearson's $r = 0.63$, $P < 0.0001$ and $r = 0.66$, $P < 0.0001$, Student's *t*-test, respectively). Depolarization duration is color coded, and the black outlined circles, indicating the means, darken with increasing depolarization duration.

H Quantification of exocytosis by whole-cell ΔC_m and single-synapse iGluSnFR-AUC. (See Materials and Methods).

I The kinetics of iGluSnFR signal. Average iGluSnFR responses (as shown in panel (D) top). Black lines indicate the results of fitting to the average traces per depolarization duration (see Materials and Methods). Time to peak and the decay time constants are obtained from these fits and depicted with the color codes of the depolarization durations.

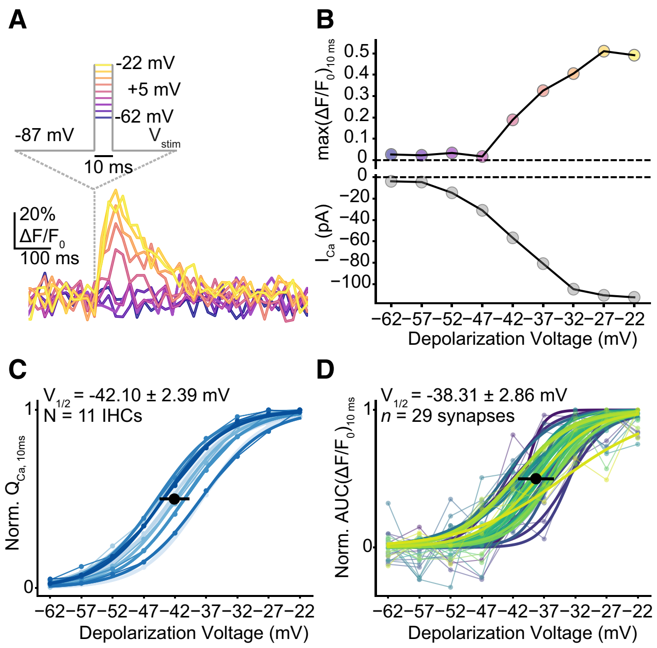

Figure EV2. Low voltage threshold for IHC glutamate release. Related to Fig 1.

A Exemplary single-synapse iGluSnFR signal in response to 10-ms-long step depolarizations from the holding potential of −87 mV to a voltage within the physiologically relevant range of receptor potentials: from −22 to −62 mV in 5 mV increments, same protocol as in Fig 1C and D.

B The peak iGluSnFR fluorescence (top; $max(\Delta F/F_0)_{10ms}$) from (A) and corresponding whole-cell Ca^{2+} current (bottom; perforated patch-clamp, 1.3 mM $[Ca^{2+}]_e$). Glutamate release can be detected at −42 mV. The voltage range is color coded: Lighter points indicate more positive potentials.

C Normalized whole-cell Q_{Ca}, calculated in response to 10-ms-long step depolarizations, is plotted as a function of depolarization voltage (perforated patch-clamp, 1.3 mM $[Ca^{2+}]_e$). Individual IHCs are color coded in shades of blue (mean ± SD, $N = 11$ IHCs from nine mice). A Boltzmann function was fitted to estimate the V_{10} and $V_{1/2}$.

D Normalized iGluSnFR-AUC, in response to 10-ms-long step depolarizations, same experiments as in (A) (mean ± SD, $n = 29$ synapses; individual synapses are color coded). A Boltzmann function was fitted to estimate the V_{10} and $V_{1/2}$.

 © 2020 The Authors

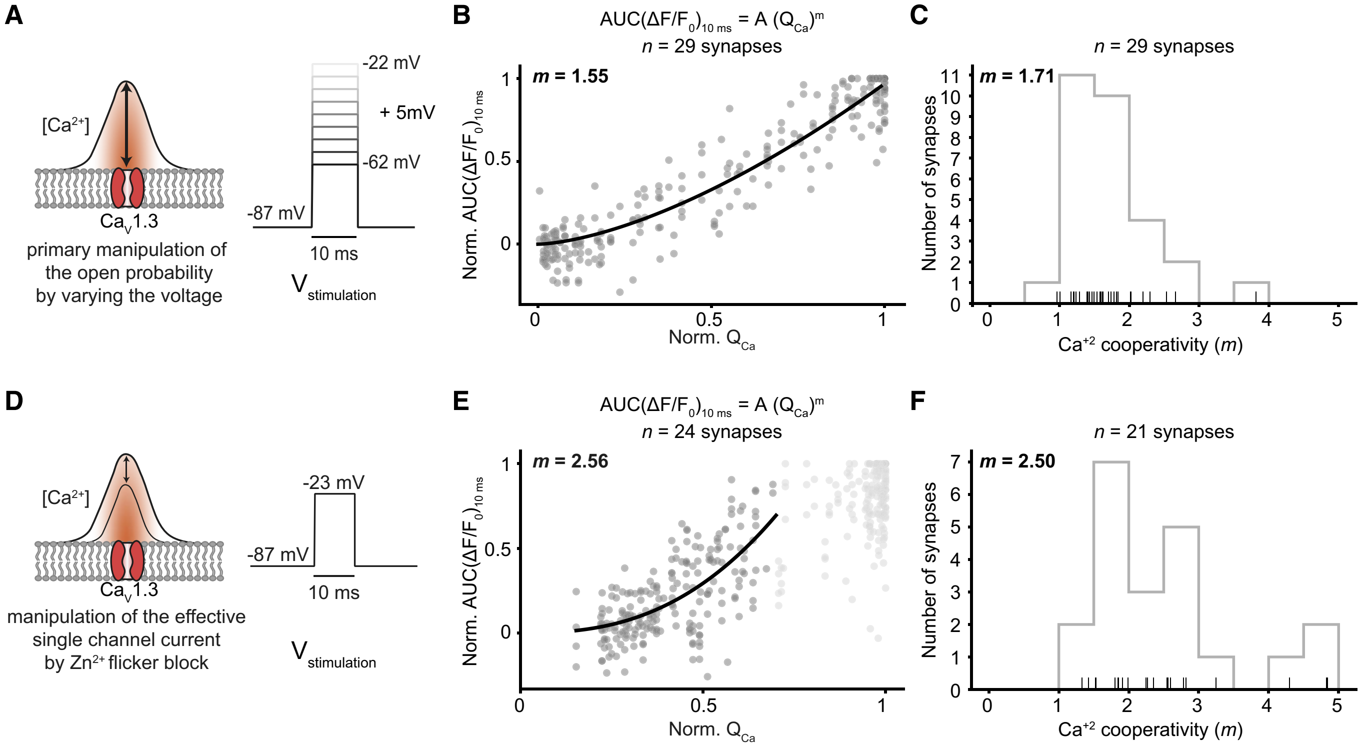

Figure EV3. Relating release to whole-cell Ca^{2+} influx during manipulation of open-channel number or single-channel current supports Ca^{2+} nanodomain-like control of release. Related to Figs 1 and 3.

- A Varying the voltage in the hyperpolarized range primarily varies the open-channel number. RRP release was probed by 10-ms-long step depolarizations from the holding potential (−87 mV) to −62 to −22 mV in 5 mV steps applied in pseudo-randomized order.
- B The normalized $AUC(\Delta F/F_0)_{10ms}$ is plotted versus Q_{Ca} (n = 29 boutons, N = 11 IHCs from nine mice): A power function was fitted before an obvious saturation of the RRP release, and a near-linear relation was observed (m = 1.55).
- C Histogram showing the distribution of m from individual fits per synapse before an obvious saturation of the RRP release (m_{avg} = 1.71 ± 0.58). Only those fits with an R^2 value higher than 0.7 were used for further analysis (n = 29 boutons). The rug plot under the histogram displays individual data points. Every depolarization step was repeated at least two times per synapse, and the average was taken.
- D Manipulation of the single Ca^{2+} channel current by Zn^{2+} perfusion. Note that Zn^{2+} acts as a rapid (microsecond) flicker blocker of Ca^{2+} channels (Winegar & Lansman, 1990), which, within the limits of IHC exocytosis kinetics (delay ~ 1 ms) (Beutner *et al*, 2001), is expected to reduce the fusogenic Ca^{2+} signal at the SV release site (Brandt *et al*, 2005). Therefore, this manipulation is expected to reveal the supralinear intrinsic Ca^{2+} dependence of release. We evoked RRP exocytosis by repetitive 10-ms-long step depolarizations to −22 mV, while slowly perfusing 1 mM Zn^{2+} into the recording chamber.
- E Normalized $AUC(\Delta F/F_0)_{10\ ms}$ is plotted versus Q_{Ca} upon Zn^{2+} manipulation (n = 24 boutons, N = 10 IHCs from 10 mice). A power function was fitted before an obvious saturation of RRP release (normalized Q_{Ca} < 0.7), and a supralinear relation was observed (m = 2.56).
- F Histogram showing the distribution of m from individual fits synapse before an obvious saturation was observed for a given synapse ($m_{average}$ = 2.50 ± 1.03, n = 21 boutons with an R^2 of fit > 0.7). The rug plot under the histogram displays the individual data points.

© 2020 The Authors

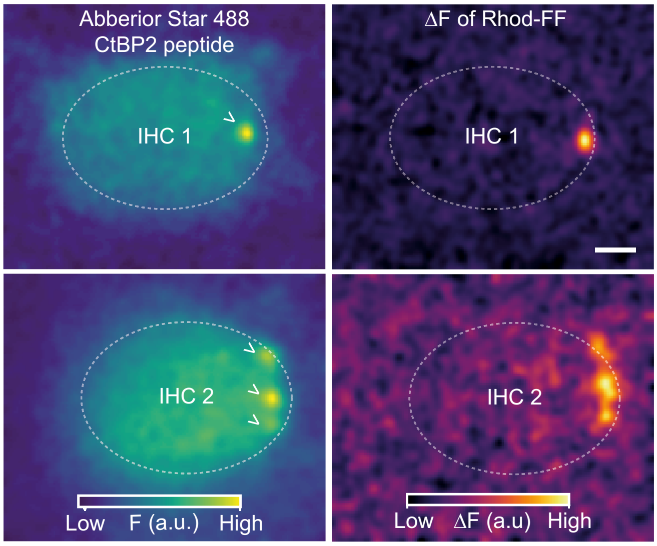

Figure EV4. Depolarization-evoked Ca^{2+} "hot spots" of Rhod-FF fluorescence occur at the AZ. Related to Fig 2.

(Left) Confocal sections of two example IHCs showing the fluorescence of the Abberior Star 488-conjugated CtBP2-binding peptide, which stains the synaptic ribbons. (Right) ΔF images of Rhod-FF in response to a 100-ms-long step depolarization to −17 mV from the holding potential of −87 mV. The color maps of the F and ΔF images are in arbitrary units (a.u.) and displayed on the bottom. (>: synaptic ribbons, scale bar: 2 μm).

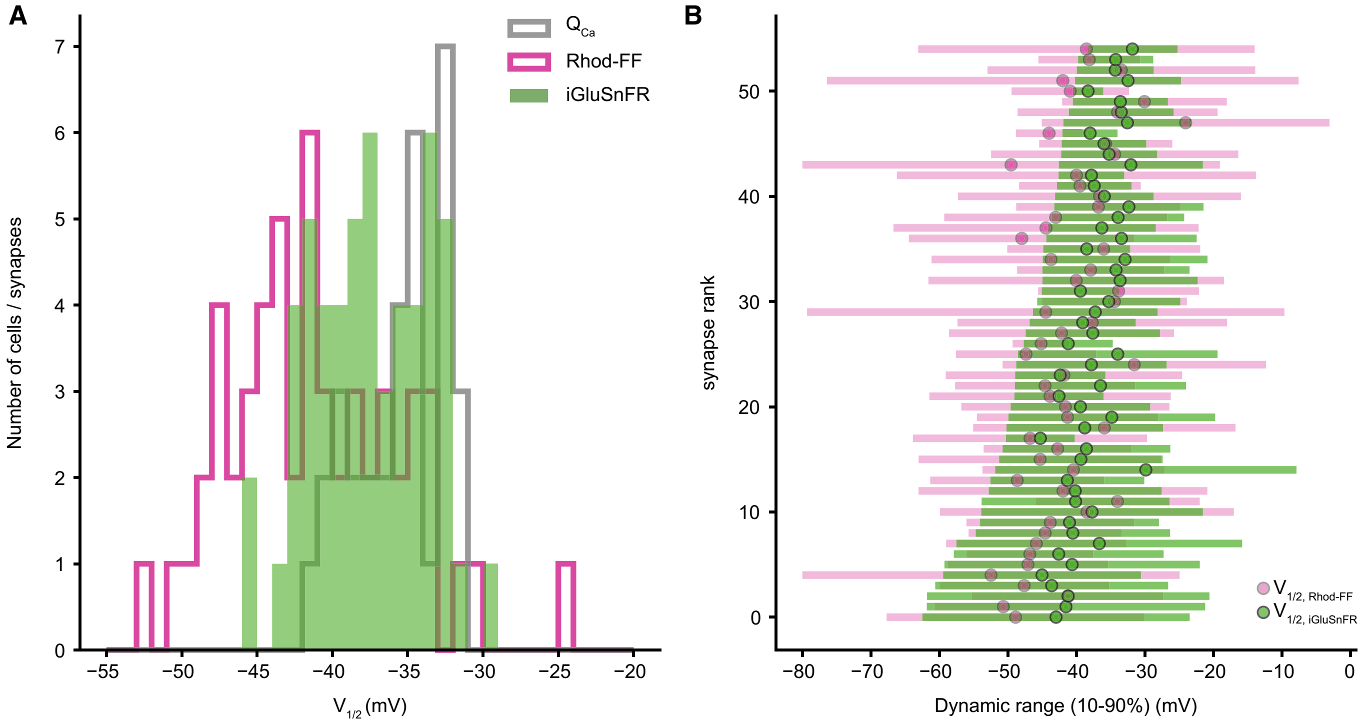

Figure EV5. AZs vary in their voltage dependence. Related to Fig 3.

A $V_{1/2}$ distribution of Q_{Ca} (gray, *N* = 34 IHCs), synaptic Ca^{2+} influx (magenta), and glutamate release (green, *n* = 55 synapses from 34 IHCs).

B Dynamic ranges (10–90%) of the synaptic Ca^{2+} influx (magenta) and glutamate release (green) with their $V_{1/2}$ depicted. The synapses are ranked based on their glutamate release threshold (V_{10}). Note how they gradually span the voltage range from −62.48 to −38.41 mV (mean ± SD = 48.27 ± 6.47 mV).

© 2020 The Authors

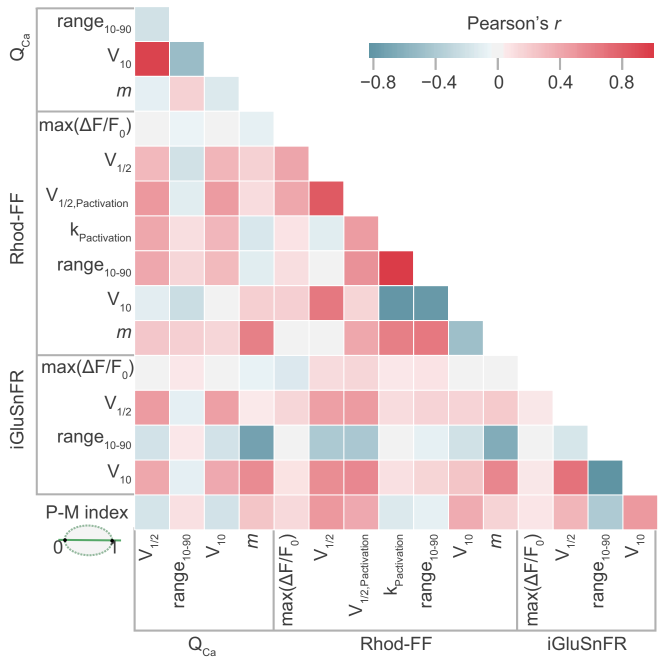

Figure EV6. Correlation map of synaptic properties. Related to Fig 3.
This correlation matrix shows the Pearson correlation coefficients (Pearson's *r*) between various properties assigned to individual synapses. The degree of correlation is color coded: light (weak) to dark (strong). Positive correlations are depicted in red and negative ones in blue.

© 2020 The Authors
